# IL-23 signaling regulation of pro-inflammatory T-cell migration uncovered by phosphoproteomics

**DOI:** 10.1371/journal.pbio.3000646

**Published:** 2020-03-23

**Authors:** Candelas Álvarez-Salamero, Raquel Castillo-González, Gloria Pastor-Fernández, Isabel R. Mariblanca, Jesús Pino, Danay Cibrian, María N. Navarro

**Affiliations:** 1 Centro de Biología Molecular Severo Ochoa, Consejo Superior de Investigaciones Científicas and Universidad Autónoma de Madrid (CSIC/UAM), Madrid, Spain; 2 Departamento de Medicina, Universidad Autónoma de Madrid, Madrid, Spain; 3 Instituto de Investigación Sanitaria del Hospital Universitario de La Princesa, Universidad Autonoma de Madrid, Madrid, Spain; Children's Hospital of Philadelphia and The University of Pennsylvania School of Medicine, UNITED STATES

## Abstract

Interleukin 23 (IL-23) triggers pathogenic features in pro-inflammatory, IL-17-secreting T cells (Th17 and Tγδ17) that play a key role in the development of inflammatory diseases. However, the IL-23 signaling cascade remains largely undefined. Here, we used quantitative phosphoproteomics to characterize IL-23 signaling in primary murine Th17 cells. We quantified 6,888 phosphorylation sites in Th17 cells and found 168 phosphorylations regulated upon IL-23 stimulation. IL-23 increased the phosphorylation of the myosin regulatory light chain (RLC), an actomyosin contractibility marker, in Th17 and Tγδ17 cells. IL-23-induced RLC phosphorylation required Janus kinase 2 (JAK2) and Rho-associated protein kinase (ROCK) catalytic activity, and further study of the IL-23/ROCK connection revealed an unexpected role of IL-23 in the migration of Tγδ17 and Th17 cells through ROCK activation. In addition, pharmacological inhibition of ROCK reduced Tγδ17 recruitment to inflamed skin upon challenge with inflammatory agent Imiquimod. This work (i) provides new insights into phosphorylation networks that control Th17 cells, (ii) widely expands the current knowledge on IL-23 signaling, and (iii) contributes to the increasing list of immune cells subsets characterized by global phosphoproteomic approaches.

## Abstract 2

Phosphoproteomics of interleukin-17-secreting T cells (Th17 cells) identifies more than 100 phosphorylation events in response to interleukin-23 stimulation, revealing increased phosphorylation of myosin regulatory light chain (RLC) and a role for an IL-23/ROCK pathway in controlling migration of Th17 and Tγδ17 cells.

## Introduction

Interleukin 23 (IL-23) is a pro-inflammatory cytokine implicated in the resolution of infections caused by particular extracellular pathogens. In addition, several reports have established a solid connection between excessive IL-23 production and the development of inflammatory diseases in murine models of experimental autoimmune encephalomyelitis (EAE), psoriasis, and inflammatory bowel disease [1–3]. Furthermore, increased levels of IL-23 have been found in biopsies from patients with Crohn disease, ulcerative colitis, and psoriasis [3,4]. This pivotal role of IL-23 in inflammatory diseases has drawn an enormous research interest in IL-23 as a therapeutic target [3].

IL-23 is formed by the p40 and p19 subunits, and it is secreted by activated dendritic cells and macrophages [5]. Its surface receptor is formed by the IL-12Rβ1 and the IL-23R chains [6]. In steady-state, IL-23R expression is restricted to minor subpopulations of TCR gamma/delta cells (TCRγδ), type 3 innate lymphoid cells (ILC3), natural killer T cells, and macrophages, and IL-23R expression is induced in CD4 T cells during the differentiation towards the Th17 helper subset [3]. IL-23 signaling triggers the production of secreted mediators such as IL-17 and IL-22, in pro-inflammatory, IL-17-secreting TCRγδ (Tγδ17), and in Th17 cells. These pro-inflammatory mediators promote the rapid recruitment and activation of granulocytes and macrophages that eventually lead to the development of clinical symptoms. The therapeutic interest of IL-23/IL-17 has led to the development of neutralizing antibodies (anti-IL-12/anti-IL-23p40 and anti-IL-23p19) for treatment of inflammatory bowel diseases and psoriasis [4,7]. Nevertheless, effective therapies for inflammatory diseases can require the combination of multiple immunomodulatory drugs to prevent disease progression and to improve quality of life. In this context, therapeutic strategies aimed at inhibiting signaling cascades have been successfully applied for treatment of immune-mediated diseases [8]. However, these strategies have not been fully exploited for IL-23-related pathologies because its signaling cascade remains largely uncharacterized. IL-23 triggers the activation of the Janus family of tyrosine kinases (JAKs) and the Signal Transducers and Activators of Transcription (STATs). IL-23 receptor is associated with JAK2 and TYK2 that promote STAT3 phosphorylation and activation [6], and active STAT3 increases the expression of the transcription factor RORγt, which is crucial for IL-17 production [9]. However, reported data suggest that in addition to the STAT3/RORγt axis, other IL-23-regulated events are involved in disease development. For example, STAT3 activation is not restricted to IL-23, because other cytokines (type I interferons, IL-6, IL-21, IL-10, or IL-27) promote STAT3 activation without triggering pathological effector functions or even exerting anti-inflammatory effects [10]. In addition, although RORγt expression is essential for the differentiation of Th17 cells, it is not required for the development of disease in EAE model [11].

The transcriptional program that controls Th17 differentiation and function and the impact of IL-23 have been extensively studied using genome-wide and even single-cell genomic approaches [12–16]. To expand the current knowledge on the IL-23 signaling cascade, we have gone one step further and characterized the phosphorylation-dependent signaling network triggered by IL-23 in primary murine Th17 cells using large-scale quantitative phosphoproteomics. We identified 6,888 phosphorylation sites comprising a substantial fraction of the Th17 phosphoproteome, and 168 phosphorylation sites showed significant changes in response to IL-23. Among the IL-23-responsive phosphosites, we focused on IL-23-induced phosphorylation of the Ser20 residue of the myosin regulatory light chain (pRLC-S20), a key regulator of actomyosin cytoskeleton contractibility and cell migration [17]. IL-23-mediated RLC phosphorylation was conserved in two IL-23-target subpopulations, the Th17 and Tγδ17 cells, and it required JAK2 and ROCK kinase activities. Moreover, our studies exploring the biological relevance of IL-23/ROCK/pRLC-S20 axis revealed an unexpected role of IL-23 in the migration of Tγδ17 and Th17 cells. To summarize, our phosphoproteomic analysis has uncovered a novel role of IL-23 in the regulation of cell motility through ROCK activation and provides a unique resource to obtain new insights on signaling cascades that can be used to generate new hypothesis-driven research. Ultimately, these data can contribute to the development of novel therapies for autoimmune and inflammatory diseases.

## Results

### Quantitative phosphoproteomics in Th17 cells

**Fig 1:**
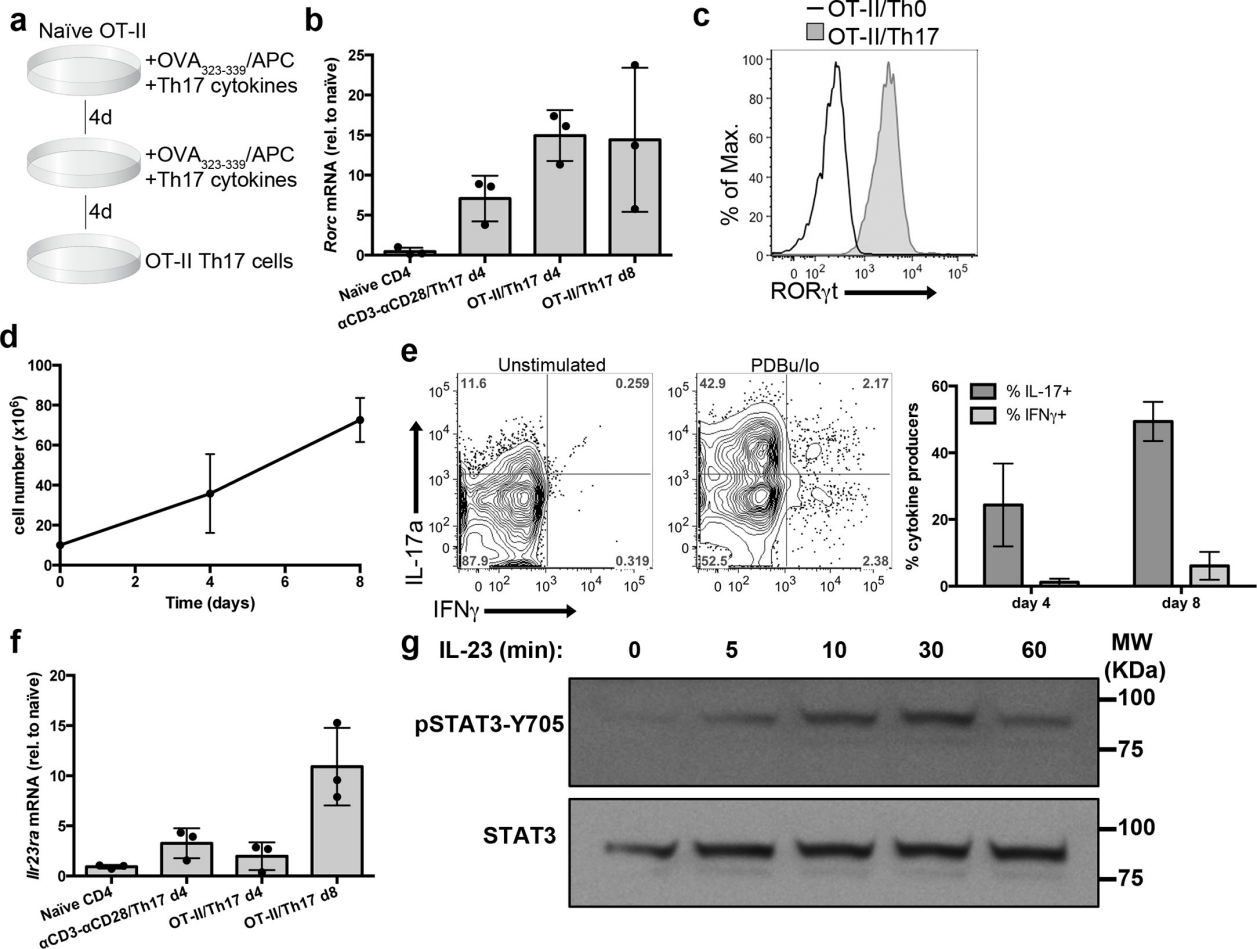
Generation of OT-II/Th17 cells. OT-II naïve CD4 T cells were co-cultured with irradiated splenocytes loaded with ovalbumin-derived peptide OVA_323-339_ as APCs, in the presence of Th17 polarizing cytokines (TGFβ, IL-6, IL-1β, IL-21, and IL-23). (a) Graphical representation of OT-II/Th17 culture protocol. (b) Graph shows *Rorc* mRNA expression in the indicated cell cultures, determined by qPCR and normalized to CD4 naïve cells (mean ± sd, *n* = 3 independent cell cultures). (c) Representative histogram shows RORγt expression in OT-II/Th0 and OT-II/Th17 cultures, determined by flow cytometry (*n* = 4 independent cell cultures). (d) Graph depicts the number of CD4 T cells at the indicated days of culture (mean ± sd, *n* = 4 independent cell cultures). (e) OT-II/Th17 cultures were stimulated with PDBu/Io or left unstimulated for 4 h in presence of Golgi-Plug, and IL-17a and IFNγ production was determined by flow cytometry. Representative contour plots show IL-17a and IFNγ production, and graph shows the percentage of cytokine-producing cells (mean ± sd, *n* = 10–11 independent cell cultures). (f) Graph shows *Il23r* mRNA expression in the indicated cell cultures, determined by qPCR and normalized to CD4 naïve cells (mean ± sd, *n* = 3 independent cell cultures). (g) Western blot analysis of STAT3-Y705 phosphorylation in response to IL-23 stimulation. Representative of *n* = 4 independent cell cultures. Individual numerical values for quantifications presented in Fig 1 can be found in S1 Data. Western blot raw images for Fig 1G can be found in S1 Raw images. APC, antigen presenting cell IFNγ, interferon gamma; IL-23, Interleukin 23; PDBu/Io, Phorbol 12,13-dibutyrate/Ionomycin; qPCR, quantitative polymerase chain reaction.

To characterize the IL-23 signaling pathway in primary cells, we have performed quantitative phosphoproteomics using murine Th17 cells differentiated in vitro as a model of IL-23R-expressing cells. To obtain Th17 cells suitable for phosphoproteomic approaches, naïve CD4 T cells from OT-II TCR transgenic mice were activated using irradiated splenocytes loaded with cognate peptide (ovoalbumin-derived peptide OVA_323-339_)_,_ as depicted in Fig 1A. Co-cultures were supplemented with Th17 polarizing cytokines (TGFβ, IL-6, IL-1β), together with IL-21 and IL-23 to induce maximal expression of IL-23R [9]. These conditions increased mRNA expression of the transcription factor RORγt (*Rorc*), master regulator of Th17 lineage (Fig 1B). The homogeneous RORγt expression pattern obtained by flow cytometry indicated that a large majority of the cells in these cultures had initiated the Th17 differentiation program (Fig 1C). In addition, we detected a substantial increase in cell numbers in these OT-II/Th17 culture conditions (Fig 1D). To verify that these cultures generated IL-17-producing cells, we determined the production of IL-17a and IFNγ in response to phorbol esters and ionomycin stimulation (PDBu/Io). At day 8, 50% of the cells produced IL-17, with a small percentage of IFNγ-secreting cells (Fig 1E). We then explored the expression of the IL-23R in these cultures, and detected an increase of IL-23R mRNA expression with maximal levels of IL-23R detected at day 8 (Fig 1F). To determine if OT-II/Th17 cells expressed a functional IL-23R, we analyzed the ability of IL-23 to induce STAT3 phosphorylation on Tyr705 residue (pSTAT3-Y705). IL-23 induced pSTAT3-Y705 in OT-II/Th17 cells, peaking at 30 min post stimulation (Fig 1G). Collectively, these data establish OT-II/Th17 cells as a suitable model to explore IL-23 signaling in primary T cells by quantitative phosphoproteomics.

**Fig 2:**
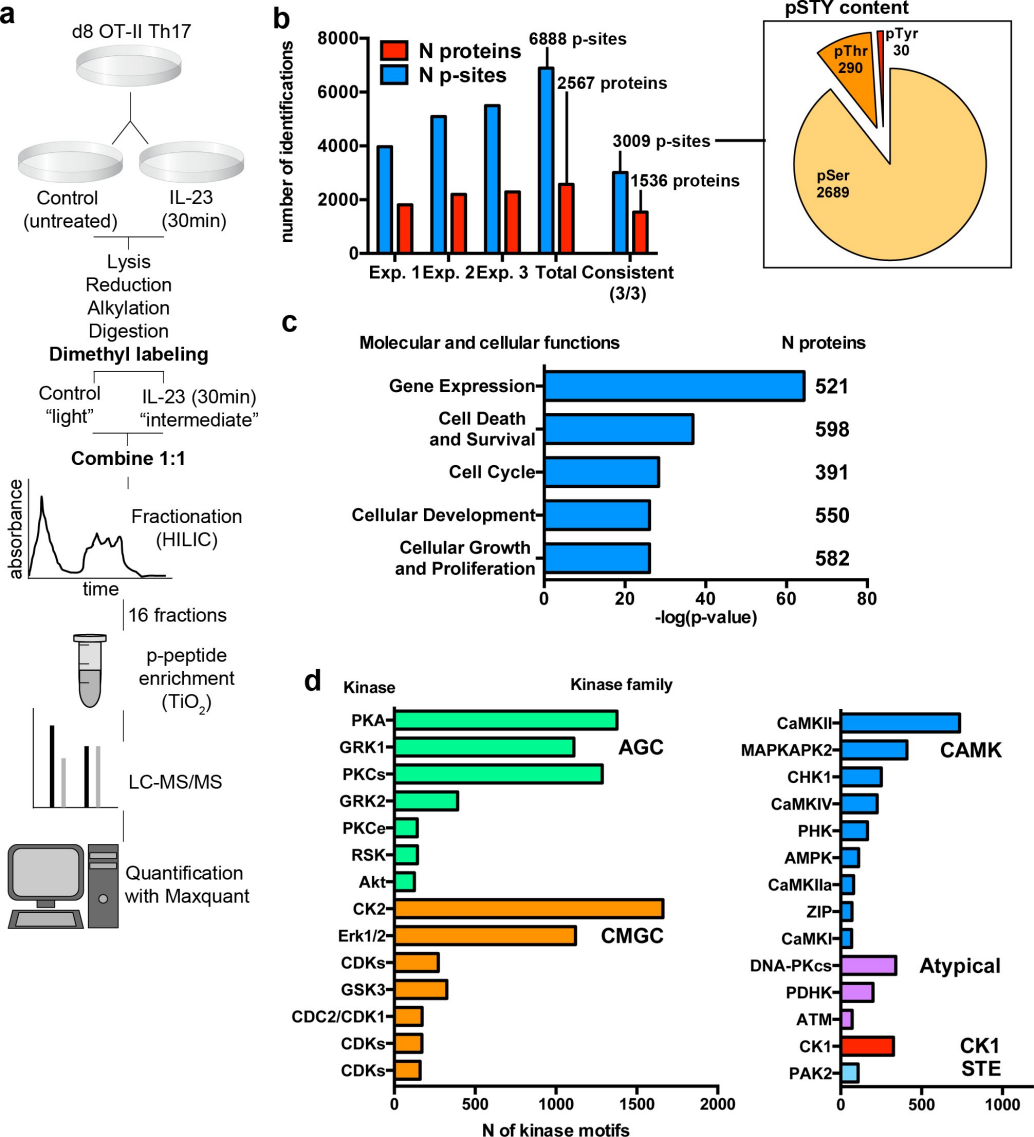
Global phosphoproteomic analysis of IL-23-stimulated OT-II/Th17 cells. (a) Experimental workflow for global phosphoproteomic analysis. (b) Graph represents the number of unique p-sites and number of distinct proteins identified and quantified in the three individual biological replicates, the collective analysis (Total), and the p-sites and proteins consistently identified in the three biological replicates. Pie chart represents the number of Serine (pSer), Threonine (pThr), and Tyrosine (pTyr) among consistently identified p-sites. (c) Graph represents the statistical significance (-log(p-value)) of the top 5 molecular and cellular functions overrepresented within the Th17 phosphoproteome. Inset numbers indicate the number of proteins included in each group. (d) Graph represents the number of potential consensus sites for the indicated kinases present in the Th17 phosphoproteome. Multiple kinase assignment to single p-site was allowed. Kinases with at least 100 consensus sites are represented. Individual numerical values for quantifications presented in Fig 2 can be found in S2 Data. IL-23, Interleukin 23; p-site, phosphorylation site.

We generated three biological replicates of OT-II/Th17 cells that were processed according to the workflow depicted in Fig 2A. Briefly, OT-II/Th17 cells were separated into two conditions at day 8 of culture: untreated control (Ctrl) and IL-23-stimulated for 30 min, in order to characterize early signaling events at a time point when maximal STAT3 phosphorylation was observed (Fig 1G). Then cells were lysed and trypsin digested. The differential dimethyl peptide labeling was used to allow the mixture of the two conditions and to avoid potential loss of material during further processing of the samples. Thus, peptides from Ctrl and IL-23 conditions were mixed at 1:1 ratio and fractionated using hydrophilic interaction liquid chromatography (HILIC). Fractions were subjected to titanium dioxide (TiO_2_) phosphopeptide enrichment and analyzed by mass spectrometry. Raw data were analyzed using the MaxQuant software that automatically performs peptide-to-protein assignment and phosphorylation-site (p-site) localization in all p-peptides identified. Moreover, for each identified p-peptide, MaxQuant generates a ratio based on the quantification of the differential dimethyl labeling (IL-23/Ctrl) that was used to determine IL-23-responsive p-sites.

The collective analysis of these experiments quantified 6,888 unique p-sites on 2,567 proteins that comprise a substantial fraction of the murine Th17 phosphoproteome, and 3,009 p-sites on 1,536 proteins were consistently identified and quantified in the three replicates (Fig 2B and S1 Table). For subsequent analysis, we focused on p-sites consistently identified in the three biological replicates. The Ser/Thr/Tyr ratio among consistent p-sites was 89/10/1, similar to other phosphoproteomes from different cell types including T lymphocytes (Fig 2B and the work by Navarro and colleagues [18]). To gain insights into the molecular and cellular functions regulated by phosphorylated proteins in Th17 cells, we subjected the phosphorylated proteins to functional pathway analysis using the Ingenuity Pathway Analysis software (IPA). IPA analysis revealed that phosphorylated proteins in Th17 cells are involved in functions such as gene expression, cell death and survival, and cell cycle (Fig 2C and S2 Table). Thus, as described for other T-cell phosphoproteomes [19,20], phosphorylation networks in Th17 cells are devoted to the maintenance of key features of effector T cell: transcription, survival, and cell division. This analysis also showed that Th17 phosphoproteome was overrepresented by proteins involved in cell development, growth, and proliferation. To identify the core kinases responsible for the Th17 phosphorylation network, we performed a kinase-substrate profiling using the motif analysis tools in Perseus software. This bioinformatic tool performs a search of consensus sites for known kinases across the whole data set. Thus, for each phosphosite, the motif analysis yields the kinase(s) that are most likely to phosphorylate a particular p-site (S1 Table). This kinase-substrate profiling show that potential substrates of AGC kinase family (mainly protein kinase A (PKA), G-protein-coupled receptor kinase 1 (GRK1) and protein kinase C (PKCs)) are abundant among the murine Th17 phosphoproteome, together with members of the CMGC family such as casein kinase 2 (CK2) and the extracellular signal-regulated kinases Erk1/2 (Fig 2D). This study can be useful to predict the potential impact of kinase inhibitor treatments on Th17 effector function, and the systematic phosphoproteomic characterization of other T-cell subpopulations will facilitate the comparison of kinase-substrate profiles to identify common and unique kinases among phosphoproteomes.

### IL-23-regulated phosphorylation network in Th17 cells

**Fig 3:**
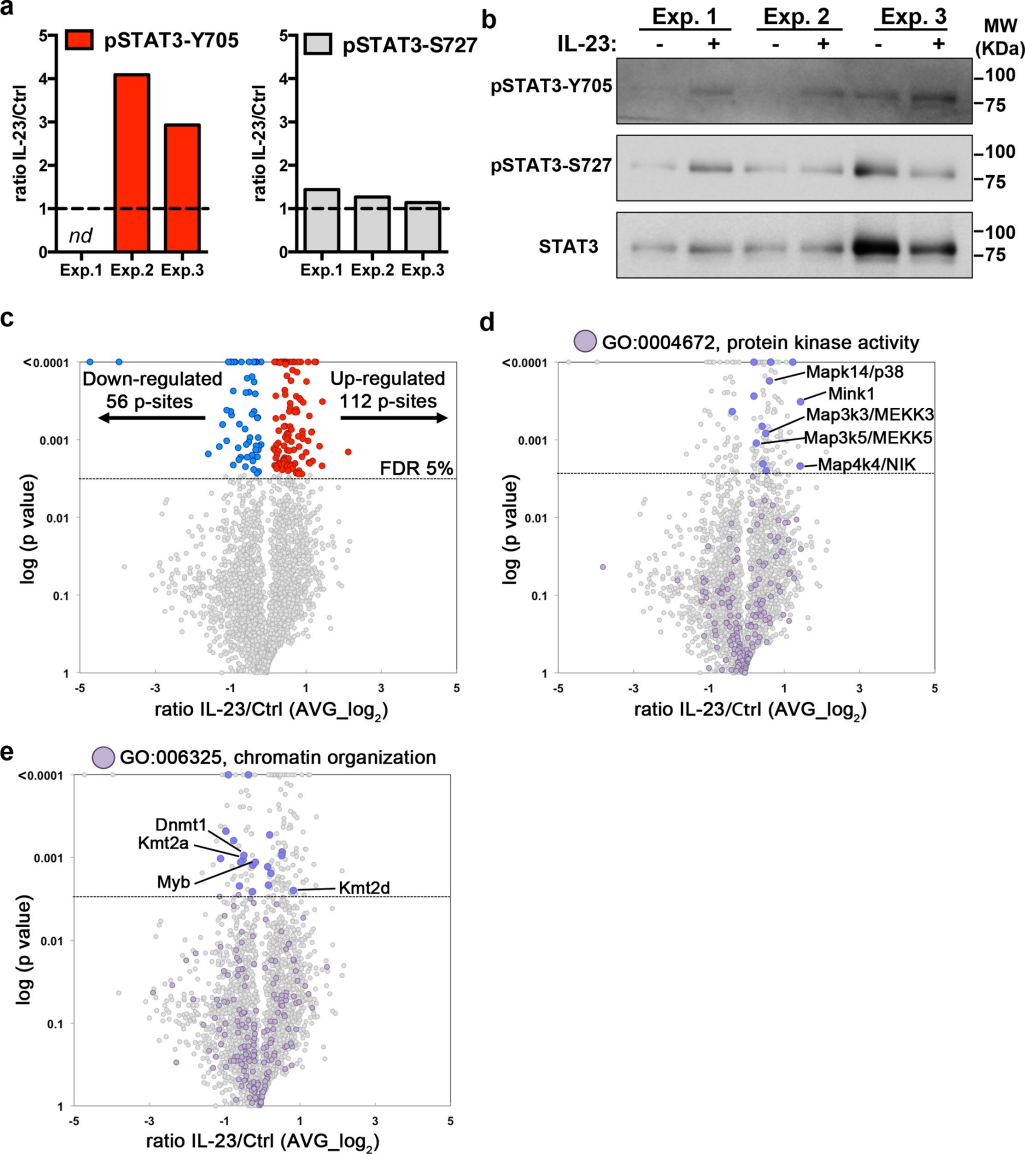
Impact of IL-23 stimulation on OT-II/Th17 phosphoproteome. (a) Graphs show the ratio IL-23/Ctrl (linear value) for the indicated STAT3 residues in the three biological replicates (nd, not determined). (b) Western blot analysis show STAT3 phosphorylation in Y705 and S727 residues in the samples used for the phosphoproteomic analysis. (c, d, e) Volcano plots show IL-23/Ctrl ratio distribution (log_2_ averaged value, AVG) against the statistical significance (–log (p value), multiple *t* test FDR 5%) for p-sites identified in the three biological replicates. Each dot represents a unique p-site. (c) Colored dots and inset numbers indicate significant IL-23-responsive p-sites (FDR 5%, red = increased and blue = decreased). (d) Colored dots indicate proteins annotated by Perseus software as protein kinases (Protein kinase activity; GO:0004672). (e) Colored dots indicate proteins annotated as chromatin remodelers (Chromatin organization; GO:006325). Individual numerical values for quantifications presented in Fig 3 can be found in S3 Data. Western blot raw images for Fig 3B can be found in S2 Raw images. Ctrl, untreated control; FDR, false discovery rate; IL-23, Interleukin 23; STAT3, Signal Transducer and Activator of Transcription family member 3.

Next, we examined the changes induced by IL-23 stimulation using the differential dimethyl labeling ratio (IL-23/Ctrl). It is well established that IL-23 induces STAT3-Y705 phosphorylation [6]. The phosphorylation on Tyrosine residues is a rare event compared to phosphorylation on Ser and Thr residues (Fig 2B and the work by Álvarez-Salamero and colleagues [21]) and thus, difficult to detect using unbiased phosphopeptide enrichment such as TiO2 affinity chromatography. Despite the low number of pTyr found in our study (Fig 2B), increased pSTAT3-Y705 was detected in the phosphoproteomic analysis in two out of the three biological replicates (Fig 3A, left graph). The increase in pSTAT3-Y705 was further validated by western blot analysis (Fig 3B). We also examined the impact of IL-23 on other key residue for STAT3 transcriptional activity, pSTAT3-S727 [22]. The quantification of pSTAT3-S727 by phosphoproteomics and western blot showed that IL-23 did not have a consistent impact on pSTAT3-S727 (Fig 3A and 3B). STAT3 phosphorylation data shown in Fig 3A and 3B demonstrated that the already characterized IL-23 signaling cascade is triggered, thus validating our phosphoproteomic analysis to uncover novel components of the IL-23 signaling network. We therefore aimed to determine the global impact of IL-23 stimulation on the murine Th17 phosphoproteome. Pairwise comparison of IL-23/Ctrl ratio among the three biological replicates showed correlation values similar to other studies using primary murine T cells (R = 0.44–0.66, S1 Fig, and the work by Ross and colleagues [23]). We restricted our analysis to the 3,009 p-sites consistently identified in the three biological replicates (Fig 2B) and detected a significant increase of 112 p-sites in 108 proteins and decreased phosphorylation of 56 p-sites in 51 proteins in response to IL-23 stimulation (Fig 3C and S3 Table). To gain insights into the phosphorylation networks regulated by IL-23, we searched for protein kinases that could be modulated by IL-23. Among the 171 annotated protein kinases in the data set, we found IL-23-responsive p-sites on 13 kinases. For example, we found increased phosphorylation of different members of the Mitogen-Activated Protein Kinase family (MAPKs): Map3k2/MEKK2, Map3k3/MEKK3, Map4k4/NIK, and Mapk14/p38α—none of them previously linked to IL-23 signaling pathway. We also found IL-23-responsive p-sites in the protein kinase Mink1 (Fig 3D). As IL-23 induction of pro-inflammatory mediators (IL-17, IL-22, etc.) is transcriptionally regulated, we searched for chromatin regulators and identified 19 p-sites that responded to IL-23 on proteins involved in chromatin organization. IL-23-responsive p-sites were found in protein methyl transferases such as Kmt2a, Kmt2d or Dnmt1, and the transcription factor Myb (Fig 3E). The functional annotations for the whole data set can be found in S1 Table. To discriminate between a potential change in protein expression rather than phosphorylation in the 168 IL-23-responsive p-sites, we analyzed all the phosphosites found in the data set for the proteins containing IL-23-responsive sites. For 96 p-sites, we could find evidence of other p-sites within the same protein that were not significantly changed by IL-23, suggesting that the protein expression was not affected. For 72 p-sites, only one or two phosphosites were found, and hence we could not extract any conclusion about their expression.

**Fig 4:**
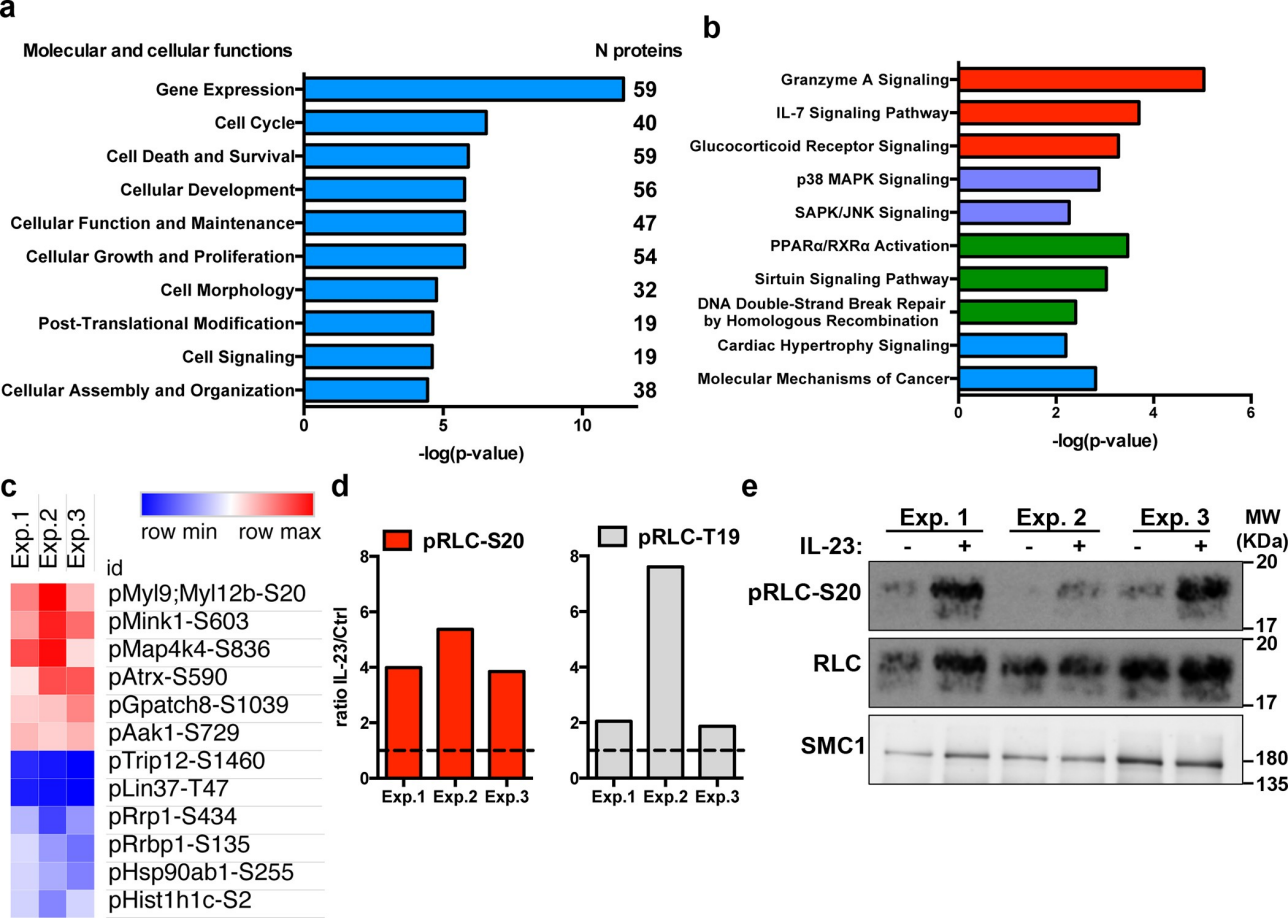
IL-23 regulated signaling pathways in the OT-II/Th17 phosphoproteome. (a) Graph represents the statistical significance (-log(p-value)) of the top 10 molecular and cellular processes overrepresented among the IL-23-responsive phosphoproteins. Inset numbers indicate the number of proteins included in each group. (b) Graph represents the statistical significance (-log(p-value)) of the top 10 canonical signaling pathways overrepresented within the IL-23-responsive phosphoproteins. (c) Heatmap represents the ratio IL-23/Ctrl (linear value) for the top significantly up-regulated and down-regulated p-sites in the individual biological replicates. (d) Graphs show the ratio IL-23/Ctrl (linear value) for the indicated residues of the RLC in the three biological replicates. (e) Western blot analysis of RLC-S20 phosphorylation in the samples used for the phosphoproteomic analysis. SMC1 was used as the loading control. Individual numerical values for quantifications presented in Fig 4 can be found in S4 Data. Western blot raw images for Fig 4E can be found in S3 Raw images. Ctrl, untreated control; IL-23, Interleukin 23; RLC, myosin regulatory light chain; SMC1, structural maintenance of chromosomes 1A.

To gain insights into the molecular and cellular functions regulated by the IL-23-responsive network, we performed IPA functional enrichment analysis with the proteins containing IL-23-responsive p-sites, including proteins with both up-regulated and down-regulated phosphorylations. IL-23-regulated phosphoproteome was found overrepresented by proteins regulating gene expression, cell cycle and survival, and also cell morphology, post-translational modifications, and signaling (Fig 4A and S4 Table). The IPA analysis of canonical signaling pathways showed that some of the IL-23-responsive proteins were involved in signaling pathways triggered by cytokine receptors and other surface receptors (i.e, Granzyme A and IL-7 receptor signaling), together with proteins involved in key signaling modules such as p38 MAPK signaling or SAPK/JNK activation (Fig 4B and S4 Table).

The top changes induced by IL-23 stimulation included p-sites on the myosin regulatory light chain (RLC, encoded by transcripts *Myl9* and *Myl12b*), three different kinases (Mink1, Map4k4/NIK, and Aak1), and the global transcriptional regulator Atrx. Among the decreased p-sites, we found the ribosomal RNA processing and ribosomal binding proteins (Rrp1 and Rrbp1), a member of the heat shock protein 90 alpha family (Hsp90ab1), and the histone H1C (Hist1h1c; Fig 4C). We focused on the top consistent change induced by IL-23: the phosphorylation of Ser20 residue on RLC. RLC is a key component of the nonmuscle myosin II complex with an essential function in the regulation of actin cytoskeleton, cell migration, and motility [17,24]. The phosphoproteomic data showed that IL-23 consistently increased the phosphorylation on RLC-Ser20 residue in the three biological replicates (Fig 4D, left graph). We also detected increased phosphorylation of RLC-Thr19 upon IL-23 stimulation, although this increase did not reach statistical significance (Fig 4D, right graph). Interestingly, the phosphorylation of RLC-T19/S20 promotes the stable association of myosin to actin filaments, forming actomyosin networks capable of generating contractile forces, and thus, pRLC-T19/S20 is widely used as an actin cytoskeleton contractibility marker [17,24]. We further validated the phosphoproteomic results by analyzing pRLC-Ser20 by western blot (Fig 4E). These results confirmed that IL-23 triggers the phosphorylation of key residues in RLC.

### IL-23 promotes RLC phosphorylation in Tγδ17 cells

**Fig 5:**
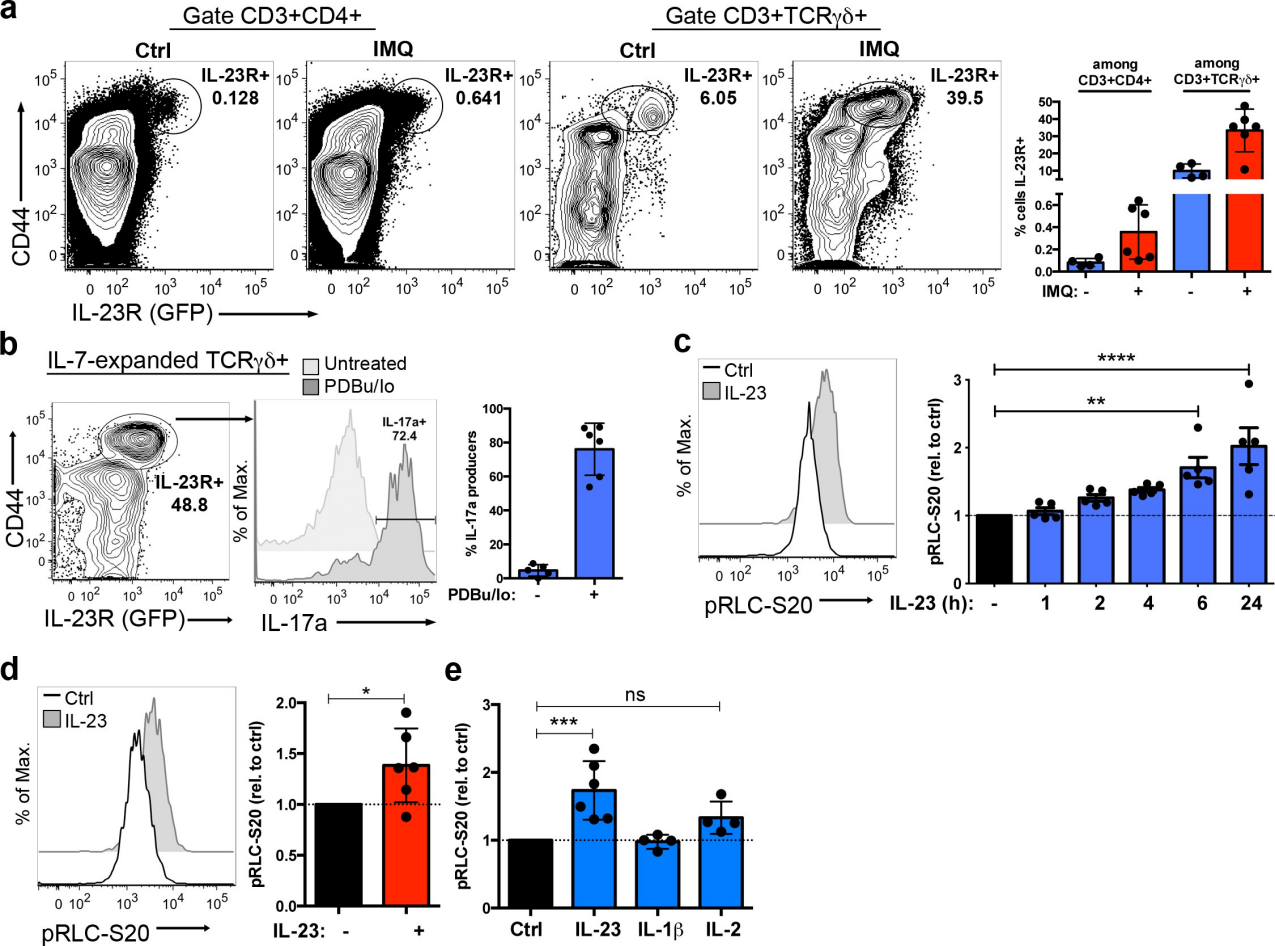
IL-23 induces RLC-S20 phosphorylation in TCRγδ IL-23R+ cells. (a) *Il23r*-gfp reporter mice were treated with IMQ or left untreated (Ctrl) for 5 days. Lymph node cells were processed for analysis of IL-23R/GFP expression by flow cytometry. Representative contour plots show CD44 and IL-23R/GFP expression in the indicated subpopulations (CD3+CD4+ and CD3+TCRγδ+), and inset numbers represent the percentage of IL-23R+ cells within the indicated gates. Graph represents the percentage of cells expressing the IL-23R (mean ± sd, *n* = 4–6 mice). (b) TCRγδ cells were isolated from lymph nodes of *Il23r*-gfp reporter mice and cultured in presence of IL-7 for 5 days. Representative contour plot shows CD44 and IL-23R (GFP) expression in IL-7-expanded TCRγδ+ cells, and inset number represents the percentage of IL-23R+ cells in the indicated gate. IL-7-expanded TCRγδ cells were stimulated with PDBu/Io in presence of Golgi-Plug or left untreated for 4 h, and IL-17a production was determined by flow cytometry. Representative histograms show IL-17a production in Tγδ17 cells (gated as CD3+TCRγδ+CD44hi), and inset number represents the percentage of IL-17a+ cells. Graph shows the percentage of IL-17a-producers among Tγδ17 cells (mean ± sd, n = 5–6 independent cultures). (c) IL-7-expanded Tγδ17 cells were stimulated with IL-23 or left untreated (Ctrl) for different time points before measuring pRLC-S20 by flow cytometry. Representative histogram shows pRLC-S20 after 6 h of IL-23 stimulation. Graph shows the MFI of pRLC-S20 in response to IL-23 at the indicated time points, normalized to MFI of untreated cells (mean ± sd, n = 5 independent cell cultures, **p = 0.0037, ****p < 0.0001). (d) Total lymph node cells obtained from IMQ-sensitized mice were stimulated ex vivo with IL-23 for 18 h or left untreated (Ctrl) before assessing pRLC-S20 by flow cytometry. Representative histogram shows pRLC-S20 in Tγδ17 (gated as CD3+TCRγδ+CD44hi). Graph shows pRLC-S20 MFI in response to IL-23, normalized to untreated controls (mean ± sd, *n* = 6 mice, *p = 0.0487). (e) IL-7-expanded Tγδ17 cells were stimulated with IL-23, IL-1β, or IL-2 for 6 h, and pRLC-S20 was determined by flow cytometry. Graph shows pRLC-S20 MFI in Tγδ17 (gated as TCRγδ+CD44hi), normalized to untreated controls (mean ± sd, n = 4–6 independent cell cultures, ***p = 0.0006). Statistical analysis: (c, e) One-way ANOVA test with Dunnett´s correction for multiple comparisons. (d) One sample *t* test. Individual numerical values for quantifications presented in Fig 5 can be found in S5 Data. Ctrl, untreated control; GFP, green fluorescent protein; IL-23, Interleukin 23; IMQ, Imiquimod; MFI, mean of fluorescence intensity; PDBu/Io, Phorbol 12,13-dibutyrate/Ionomycin; pRLC-S20, phospho-RLC-Serine20; RLC, myosin regulatory light chain.

To further explore the IL-23/pRLC-S20 signaling network in IL-23R-expressing cells in vivo, we took advantage of IL-23R/green fluorescent protein (GFP) reporter mice [25]. As previously described [25,26], the frequency of IL-23R/GFP-expressing cells was greater among CD3+TCRγδ+ cells than among CD3+CD4+ cells, and IL-23R expression was restricted to cells expressing high levels of the activation and memory marker CD44 (Fig 5A). Moreover, sensitization with the inflammatory agent Imiquimod (IMQ), which induces a skin inflammation with common features to psoriasis in an IL-23-dependent manner [2], increased the frequency of IL-23R-expressing TCRγδ cells [27], while inducing a small increase of the CD4+IL-23R+ subpopulation (Fig 5A). The CD3+TCRγδ+IL-23R+ subpopulation, known as Tγδ17 or γδ17 T cells [28], secreted large amounts of IL-17 both in the steady-state and in IMQ-sensitized animals (S2A Fig). In addition to IL-17, the Tγδ17 subpopulation produce IL-22 and other pro-inflammatory cytokines very early during the immune response, creating an inflammatory milieu that amplify Th17 effector function [29,30]. The endogenous expression of the IL-23R confers Tγδ17 cells with an innate-like ability to respond to IL-23 before Th17 differentiation occurs [26,31]. To explore whether IL-23 regulation of RLC phosphorylation was conserved in Tγδ17 cells, we took advantage of previous work reporting that Tγδ17 cells respond to IL-7 [32]. IL-7Rα expression was higher in the TCRγδ+IL-23R+ than in TCRγδ+IL-23R- cells, and we were able to isolate TCRγδ cells from lymph nodes and expand them in vitro in presence of IL-7 (S2B and S2C Fig). These IL-7-expanded TCRγδ cells maintained Tγδ17 hallmarks such as high levels of expression of CD44, the IL-23R, and the IL-17 production capability (Fig 5B). The kinetic analysis of the impact of IL-23 stimulation showed that IL-23 gradually increased pRLC-S20, with statistical significant reached at 6 to 24 h of stimulation (Fig 5C). Thus, further studies were performed at 6 to 24 h post stimulation with IL-23. In addition to IL-7-expanded Tγδ17 isolated from naïve, nonimmunized animals, we analyzed pRLC-S20 in Tγδ17 obtained from IMQ-sensitized animals and detected a significant increase of pRLC-S20 in response to IL-23 in IMQ-primed-Tγδ17 (Fig 5D). Thus, our results show that the IL-23 signaling pathway promoting RLC-S20 phosphorylation is conserved in both naïve and pathogenic Tγδ17 cells (Fig 5C and 5D). Next, we asked the question of how selective was the IL-23 effect on RLC phosphorylation. In addition to the IL-23R, Tγδ17 express the pro-inflammatory cytokine receptors IL-1R1 [31] and IL-2Rα [26], and the expression of these markers is maintained in IL-7-expanded Tγδ17 cells (S2D Fig). The stimulation with IL-1β did not induce pRLC-S20 in IL-7-expanded Tγδ17, whereas IL-2 induced a slight but nonsignificant increase in RLC phosphorylation (Fig 5E). All these data support that pRLC-S20 is selectively induced by IL-23 in Tγδ17 cells.

### IL-23-mediated RLC phosphorylation depends on JAK2 and ROCK activity

**Fig 6:**
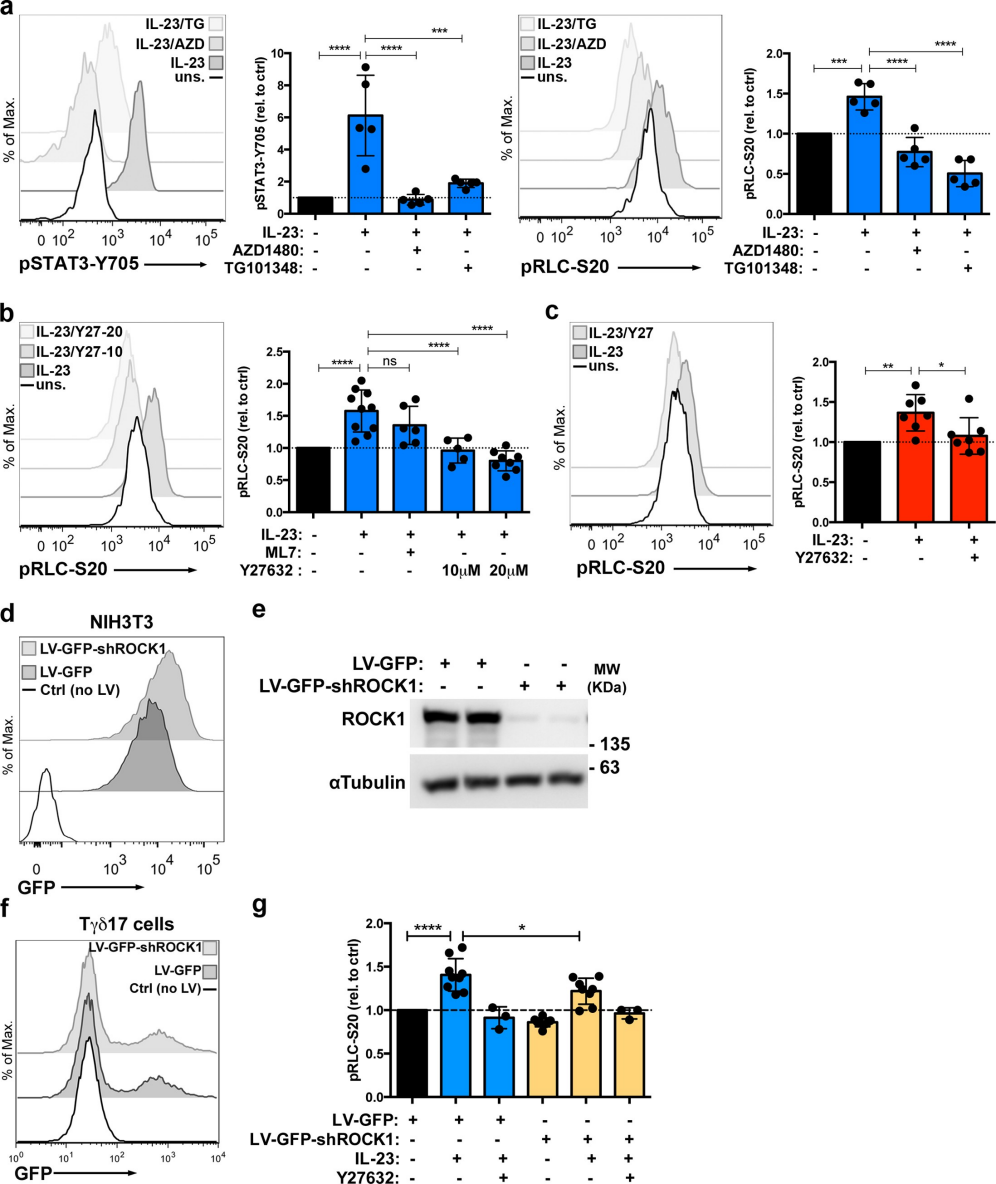
IL-23 induces pRLC-S20 through JAK2 and ROCK dependent mechanisms in Tγδ17 cells. (a) IL-7-expanded Tγδ17 cells were stimulated with IL-23 in presence or absence of the indicated JAK2 inhibitors or left unstimulated (uns.) for 6 h before assessing pSTAT3-Y705 and pRLC-S20 by flow cytometry. Representative histograms show pSTAT3-Y705 (left) and pRLC-S20 (right) in the indicated conditions. Graphs show the MFI of pSTAT3-Y705 (***p = 0.0002, ****p < 0.0001) and pRLC-S20 (***p = 0.0004, ****p < 0.0001) in TCRγδ+CD44hi cells, normalized to unstimulated controls (mean ± sd, *n* = 5 independent cultures). (b) IL-7-expanded Tγδ17 cells were stimulated with IL-23 in presence or absence of ML7 (MLCK inhibitor) or Y27632 (ROCK inhibitor) or left untreated for 6 h, before measurement of pRLC-S20 by flow cytometry. Representative histograms show pRLC-S20 in the indicated conditions. Graph shows pRLC-S20 MFI in TCRγδ+CD44hi cells, normalized to unstimulated controls (mean ± sd, n = 5–10 independent cell cultures, ****p < 0.0001). (c) Lymph node cells obtained from IMQ-sensitized mice were stimulated ex vivo with IL-23 for 18 h in presence or absence of ROCK inhibitor Y27632 or left unstimulated. Representative histogram shows pRLC-S20 in Tγδ17 (gated as CD3+TCRγδ+CD44hi). Graph shows pRLC-S20 MFI in response to IL-23 (relative MFI, mean ± sd, *n* = 7 mice, *p = 0.017, **p = 0.0031). (d, e) NIH3T3 cells were transduced with lentiviral particles LV-GFP, LV-GFP-shROCK1, or not transduced (Ctrl), and 5 days later, processed for analysis by flow cytometry and western blot. (d) Representative histograms show GFP expression (*n* = 2 independent experiments). (e) Representative western blots of ROCK1 expression in transduced NIH3T3 α Tubulin was used as loading control (*n* = 2 independent experiments). (f, g) Tγδ17 cells were transduced with LV-GFP, LV-GFP-shROCK1, or not transduced (Ctrl) and 7 days later were analyzed by flow cytometry. (f) Representative histograms show GFP expression (*n* = 8 independent experiments). (g) Transduced Tγδ17 cells were stimulated with IL-23 in presence or absence of ROCK inhibitor Y27632 or left untreated for 18 h before assessing pRLC-S20 by flow cytometry. Graph shows pRLC-S20 MFI in Tγδ17 GFP+ cells, normalized to the untreated control (mean ± sd, *n* = 8, ****p < 0.0001, *p = 0.013). Statistical analysis by one way ANOVA with Dunnet correction for multiple comparisons. Individual numerical values for quantifications presented in Fig 6 can be found in S6 Data. Western blot raw images for Fig 6E can be found in S4 Raw images. Ctrl, untreated; IL-23, Interleukin 23; IMQ, Imiquimod; JAK2, Janus kinase 2; LV-GFP, lentiviral construct encoding a GFP reporter marker; LV-GFP-shROCK1, lentiviral construct encoding a GFP reporter marker and shRNAs against ROCK1; MFI, mean of fluorescence intensity; pRLC-S20, phospho-RLC-Serine20; ROCK, Rho-associated protein kinase.

To further characterize this novel IL-23-mediated pRLC-S20, we investigated the molecular mechanisms responsible for this phosphorylation. Given that IL-23 signaling cascade is initiated by JAK2 and TYK2 activation [6], we first examined the impact of JAK2 inhibitors on IL-23-mediated pRLC-S20. IL-7-expanded Tγδ17 were treated with two different JAK2 inhibitors: AZD1480 and TG101348. At concentrations that inhibited IL-23-mediated pSTAT3-Y705 (Fig 6A, left), both JAK2 inhibitors abolished IL-23-induced pRLC-S20 (Fig 6A, right). These experiments suggested that the tyrosine kinase JAK2 controls the activation of a serine/threonine kinase responsible for the direct phosphorylation of pRLC-S20. In this context, RLC-T19/S20 residues are known substrates for the myosin light chain kinase (MLCK). In addition to MLCK, the Rho-associated protein kinase (ROCK) controls the phosphorylation status of the RLC-T19/S20 residue by direct phosphorylation, or indirectly, through the inhibition of the myosin Phosphatase-Targeting Subunit 1 (MYPT-1) [17]. We examined the impact of the reference inhibitors ML7 (MLCK inhibitor) and Y27632 (ROCK inhibitor) on IL-23-mediated pRLC-S20. Fig 6B shows that ML7 did not consistently inhibit pRLC-S20, and the treatment with Y27632 completely blocked RLC phosphorylation in response to IL-23. Moreover, we determined that IL-23-induced RLC-S20 phosphorylation was abolished in presence of ROCK inhibitor in IMQ-primed-Tγδ17 cells (Fig 6C). To further examine the role of ROCK in IL-23-induced pRLC-S20, we aimed to silence ROCK1 expression using shRNAs (shROCK1). First, we tested the silencing efficacy of a validated shROCK1 that was delivered to the mouse fibroblast cell line NIH3T3 using lentiviral constructs with a GFP reporter marker (LV-GFP-shROCK1 and control vector LV-GFP). In these experiments, high levels of expression of the GFP reporter (Fig 6D) correlated with the efficient silencing of ROCK1 expression in NIH3T3 cells (Fig 6E). Next, we transduced IL-7-expanded Tγδ17 cells with the LV-GFP-shROCK1 or LV-GFP lentiviral particles. The transduction efficiency in Tγδ17 cells was lower than in NIH3T3 cells (17% ± 7% of GFP+ cells, mean ± sd, with both control and shROCK1 lentivirus, compared to 100% efficiency in NIH3T3), and the levels of expression of the GFP reporter were substantially lower in Tγδ17 than in NIH3T3 cells (compare Fig 6D and 6F). Thus, although the numbers of transduced Tγδ17 cells did not allow direct assessment of shROCK1 silencing efficacy by western blot, the level of expression of GFP reporter suggested that the ROCK1 silencing was probably less efficient in Tγδ17 than in NIH3T3 cells. Nevertheless, we stimulated the transduced Tγδ17 cells with IL-23 and measured the impact of shROCK1 on pRLC-S20 in GFP-expressing cells by flow cytometry. As shown in Fig 6G, the IL-23-induced increase in RLC-S20 phosphorylation was significantly lower in shROCK1-transduced cells compared to the empty vector. The addition of Y27632 inhibitor further decreased pRLC-S20 in shROCK1-transduced cells, indicating that some remaining ROCK activity was still present in these cells. Thus, despite the silencing efficacy of the shROCK1 in Tγδ17 cells is likely to be low, the significant decrease in IL-23-induced pRLC-S20 in these conditions supports the participation of ROCK function in this novel signalling pathway. Overall, data in Fig 6 shows that IL-23 promotes pRLC-S20 through ROCK activation in both naïve and pathogenic Tγδ17 cells.

**Fig 7:**
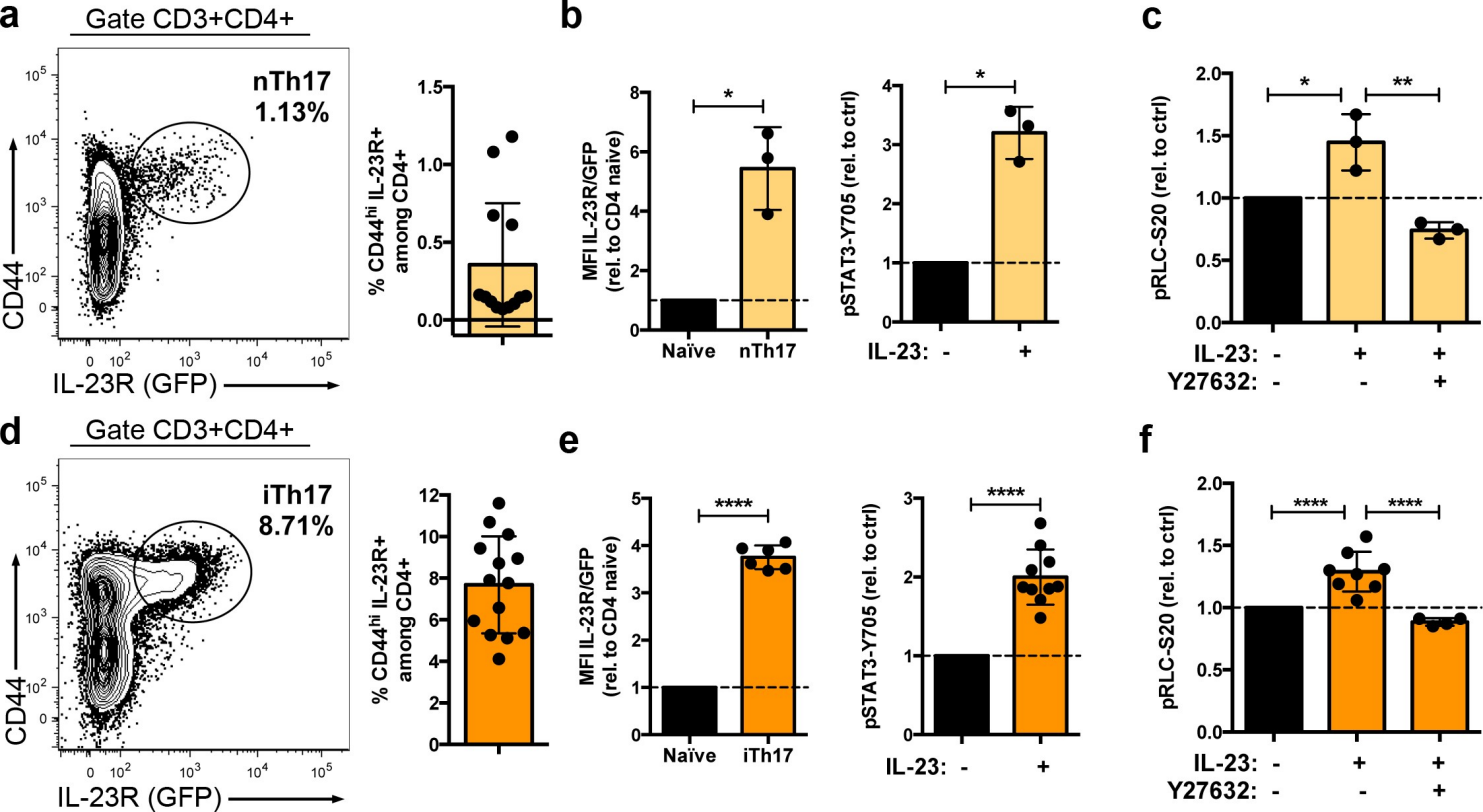
IL-23 induces pRLC-S20 through ROCK dependent mechanisms in Th17 cells. (a) Total lymph node cells from *Il23r*-gfp reporter mice were processed for analysis of IL-23R (GFP) expression by flow cytometry. Representative contour plots show CD44 and IL-23R/GFP expression in the CD3+CD4+ population, and inset number represents the percentage of IL-23R+ cells within the indicated gate. Graph represents the percentage of nTh17 cells, gated as CD3+CD4+CD44^hi^IL-23R+ (mean ± sd, *n* = 13 mice). (b) nTh17 cells were sorted from lymph nodes of *Il23r*-gfp reporter mice as CD4+CD44^hi^IL-23R+ and cultured in presence of IL-7 for 7 days. Right graph shows IL-23R/GFP MFI after 7 days of culture, normalized to CD4 naïve cells (sorted in parallel to nTh17 as CD4+CD44^low^IL-23R- and cultured for 7 days in IL-7, mean ± sd, *n* = 3 independent cultures, *p* = 0.0314). IL-7-cultured nTh17 were stimulated with IL-23 or left untreated for 18 h before assessing pSTAT3-Y705 by flow cytometry. Left graph shows pSTAT3-Y705 MFI, normalized to untreated cells (mean ± sd, *n* = 3 independent cultures, *p* = 0.0132). (c) IL-7-cultured nTh17 were stimulated with IL-23 in presence or absence of Y27632 or left untreated for 18 h before assessing pRLC-S20 by flow cytometry. Left graph shows pRLC-S20 MFI, normalized to untreated cells (*n* = 3 independent cultures, *p = 0.0121, **p = 0.0012). (d) *Il23r*-gfp reporter mice were immunized with CFA/MOG to induce EAE; 12 days after EAE induction, total splenic cells were processed for analysis of IL-23R/GFP expression by flow cytometry. Representative contour plots show CD44 and IL-23R/GFP expression in the CD3+CD4+ population, and inset numbers represent the percentage of IL-23R+ cells within the indicated gate. Graph represents the percentage of iTh17 cells, gated as CD3+CD4+CD44^hi^IL-23R+ (mean ± sd, n = 14 mice from 2 independent experiments). (e) iTh17 cells were sorted from lymph nodes and spleens of EAE-treated *Il23r*-gfp reporter mice as CD4+CD44^hi^IL-23R+ and cultured in presence of IL-7 for 7 days. Right graph shows IL-23R/GFP MFI after 7 days of culture, normalized to CD4 naïve cells (sorted as CD4+CD44^low^IL-23R- and cultured for 7d in IL-7, mean ± sd, *n* = 6 independent cultures, *p* < 0.0001). IL-7-expanded iTh17 were stimulated with IL-23 or left untreated for 18 h before assessing pSTAT3-Y705 by flow cytometry. Left graph shows pSTAT3-Y705 MFI, normalized to untreated cells (mean ± sd, *n* = 10 independent cultures, *p* < 0.0001). (f) IL-7-expanded iTh17 were stimulated with IL-23 in presence or absence of Y27632 or left untreated for 18 h before assessing pRLC-S20 by flow cytometry. Left graph shows pRLC-S20 MFI, normalized to untreated cells (mean ± sd, n = 4–8 independent cultures, ****p < 0.0001). Statistical analysis: (b, e) One sample *t* test. **(**c, f) One-way ANOVA test with Dunnett’s correction for multiple comparisons. Individual numerical values for quantifications presented in Fig 7 can be found in S7 Data. CFA, Complete Freunds´ Adjuvant; MOG, Myelin oligodendrocyte glycoprotein; EAE, experimental autoimmune encephalomyelitis; GFP, Green Fluorescent Protein; IL-23, Interleukin 23; iTh17, induced Th17; MFI, Mean of fluorescence intensity; nTh17, natural Th17; pRLC-S20, phospho-RLC-Serine20; ROCK, Rho-associated protein kinase.

Next, we explored if ROCK activity was required for IL-23-induced pRLC-S20 in other key IL-23-target population, the Th17 cells. We performed experiments in two different subpopulations of Th17 cells: the natural Th17 (nTh17) and the induced Th17 (iTh17) in the experimental autoimmune encephalomyelitis (EAE), a murine model of multiple sclerosis [30]. The nTh17 cells represent a minor fraction of the CD4 T cell memory-like compartment with the potential to secrete IL-17a (S3A Fig and the work by Aggarwal and colleagues [33]). In addition to the activation and memory marker CD44, the nTh17 cells express the IL-23R (Fig 7A and S3B Fig). The nTh17 cells were isolated by cell sorting as CD4+CD44hiIL-23R+ and cultured in presence of IL-7. The in vitro cultured nTh17 maintained the expression of the IL-23R and responded to IL-23 by inducing the phosphorylation of STAT3-Y705 residue (Fig 7B). IL-23 stimulation in nTh17 also induced a significant increase of pRLC-S20. Moreover, this increase was abolished by the treatment with the ROCK inhibitor Y27632 (Fig 7C). To determine whether the IL-23/ROCK/pRLC axis was conserved in pathogenic Th17 cells, we took advantage of the EAE model. This model induces the generation of antigen-specific CD4 T cells that secrete IL-17a (S3C Fig and the work by Langrish and colleagues and the work by Komiyama and colleagues [34,35]). In addition, EAE induction increased in the frequency of IL-23R-expressing CD4 T cells (compare Fig 7A and Fig 7D) that are critical for the development of the disease [1,25]. We sorted the induced Th17 cells (iTh17) as CD4+CD44hiIL-23R+ and expanded them in vitro in the presence of IL-7 (S3D Fig). The in vitro cultured iTh17 retained the ability to secrete IL-17a (S3E Fig) and maintained the expression of a functional IL-23R that induced pSTAT3-Y705 residue in response to IL-23 (Fig 7E). IL-23 stimulation in these cells also induced an increase of pRLC-S20 that was abolished by ROCK inhibitor treatment (Fig 7F). Overall, these experiments show that IL-23-induced pRLC-S20 was dependent on JAK2 and ROCK kinase activity and that the IL-23/ROCK/pRLC-S20 signaling node is conserved in both naïve and pathogenic Tγδ17 and Th17 cells.

### IL-23 promotes Tγδ17 and Th17 migration via ROCK activation

**Fig 8:**
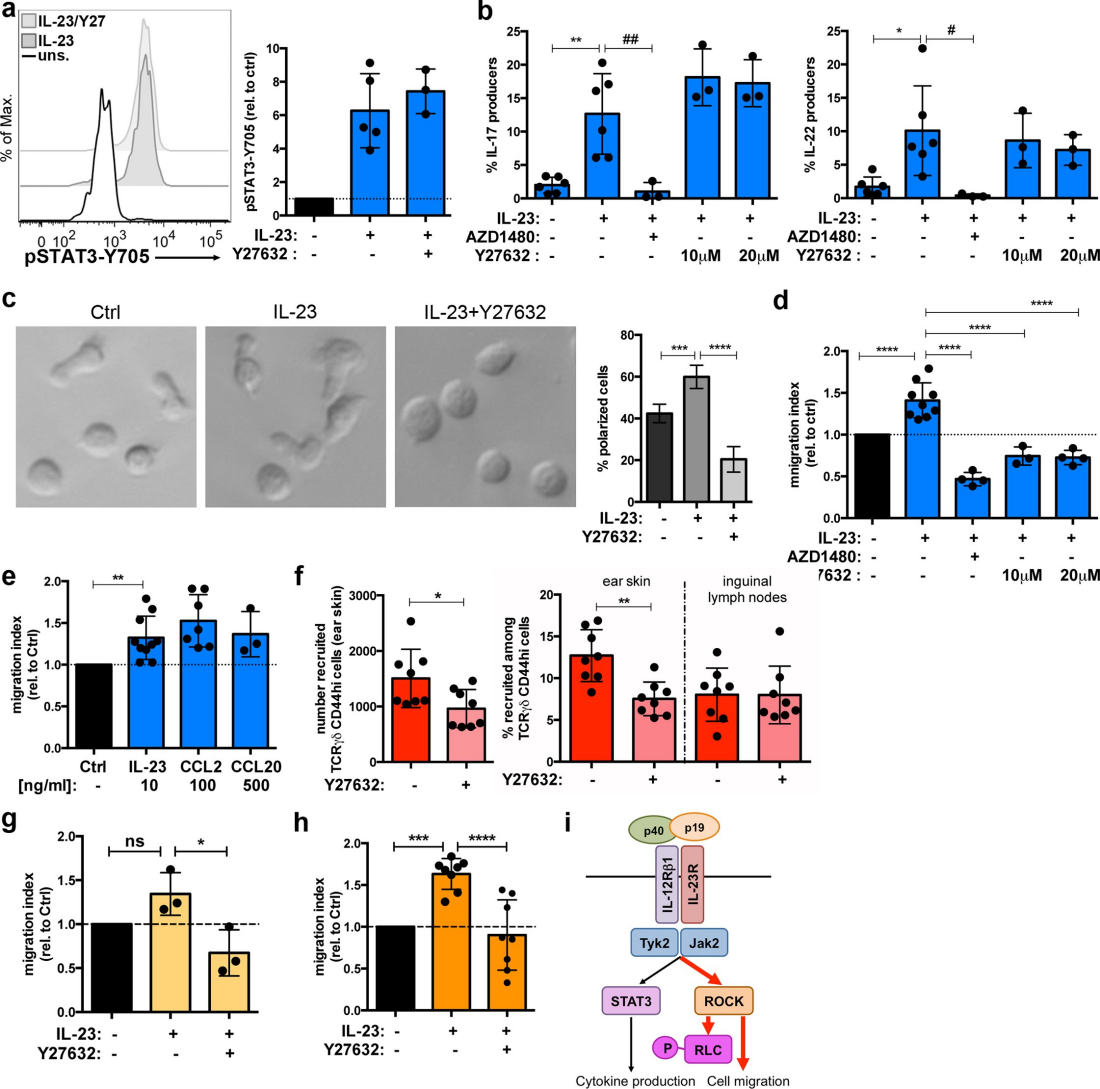
IL-23-mediated ROCK activation promotes Tγδ17 cell migration. (a) IL-7-expanded Tγδ17 cells were stimulated with IL-23 for 6 h in the presence or absence of Y27632 inhibitor or left unstimulated (uns.). Representative histogram shows pSTAT3-Y705 measured by flow cytometry. Graph shows pSTAT3-Y705 MFI in TCRγδ+CD44hi cells, relative to unstimulated cells (mean ± sd, *n* = 3–5 independent cell cultures). (b) IL-7-expanded Tγδ17 cells were stimulated with IL-23 for 18 h in the presence or absence of the indicated inhibitors or left unstimulated. Cytokine production was assessed by flow cytometry. Graphs show the percentage of IL-17a producers (left) and IL-22 (right) among TCRγδ+CD44hi cells (mean ± sd, *n* = 3–6 independent cell cultures). Left graph, **p = 0.001, ^##^*p* = 0.0028. Right graph *p = 0.0106, ^#^*p* = 0.0157. (c) IL-7-expanded Tγδ17 cells were shorted as CD27 negative and stimulated with IL-23 for 18 h or left unstimulated. Cells were allowed to settle onto fibronectin/poly-L-Lysine-coated wells for 1 h in presence of 20 μM Y27632 inhibitor before imaging. Graph shows the frequency of cells with polarized morphology (***p = 0.0001, ****p < 0.0001). Images and quantification are representative of *n* = 2 independent cell cultures. (d) IL-7-expanded Tγδ17 cells from *Il23r*-gfp reporter mice were stimulated with IL-23 for 18 h or left unstimulated. Next day cells were pretreated for 1 h with AZD1480 or Y27632 and placed into a 3-μM transwell insert. Cells were left to migrate into the lower chamber containing medium with the same conditions as the upper chamber (IL-7, ±IL-23, and ±inhibitors) for 6 h. Cell numbers in the lower chamber were determined by flow cytometry. Graph represents migrated cells (IL-23R+/GFP), relative to untreated cells (migration index, mean ± sd, *n* = 4–9 independent cultures, ****p < 0.0001). (e) IL-7-expanded Tγδ17 cells from *Il23r*-gfp reporter mice were stimulated with IL-23 for 18 h or left unstimulated. Next day, IL-23-stimulated cells were placed into a 3-μM transwell insert and left to migrate into the lower chamber containing medium with the same conditions as the upper chamber (IL-7 and IL-23). For chemotactic assays, unstimulated cells were placed in transwell inserts and left to migrate for 6 h into the lower chamber containing the indicated concentrations of chemokine ligands CCL2 or CCL20. Cell numbers in the lower chamber were determined by flow cytometry. Graph represents migrated cells (IL-23R+/GFP) relative to untreated cells (migration index, mean ± sd, *n* = 3–10 independent cultures, **p = 0.0035). (f) TCRγδ cells were isolated from 5 d IMQ-treated mice, labeled with CellTrace Violet, and adoptively transferred into recipient mice that were previously ear-treated with IMQ for 5 days. Recipient animals received 2 intraperitoneal injections of ROCK inhibitor Y27632 or control vehicle (DMSO/PBS) prior to adoptive transfer of CellTrace-labeled TCRγδ cells; 12 h after transfer, ear skin and LN from recipient mice were harvested and processed for analysis by flow cytometry in presence of Accucheck counting beads to determine numbers and frequencies of adoptively transferred Tγδ17. In skin, the adoptively transferred Tγδ17 cells were gated as dermal TCRγδ cells (CD3+TCRγδintCD44hi and CellTrace+). In LN, adoptively transferred Tγδ17 cells were gated as CD3+TCRγδ+CD44hi and CellTrace+. Left graph represents the number of recruited Tγδ17 cells to the inflamed ear skin in IMQ-sensitized mice. Right graph represents the frequency of recruited Tγδ17 cells in inflamed ear skin and inguinal lymph nodes (nondraining) of IMQ-treated mice among total Tγδ17 cells. Data are pooled from 2 experiments (*n* = 3–5 mice per group, *p = 0.03, **p = 0.002). (g) IL-7-cultured nTh17 cells from *Il23r*-gfp reporter mice were stimulated with IL-23 for 18 h or left untreated. Next day cells were pretreated for 1 h with Y27632 and placed into a 3-μM transwell insert. Cells were left to migrate into the lower chamber containing medium with the same conditions as the upper chamber (IL-7, ±IL-23, and ±inhibitor) for 2 h. Cell numbers in the lower chamber were analyzed by flow cytometry in presence of Accucheck counting beads. Graph represents migrated cells (CD4+CD44^hi^IL-23R+/GFP) relative to untreated cells (migration index, mean ± sd, *n* = 3 independent cultures, *p = 0.013). (h) IL-7-expanded iTh17 from EAE-treated *Il23r*-gfp reporter mice were stimulated with IL-23 for 18 h or left untreated. Next day cells were pretreated for 1 h with Y27632 and placed into a 3-μM transwell insert. Cells were left to migrate into the lower chamber containing medium with the same conditions as the upper chamber (IL-7, ±IL-23, and ±inhibitor) for 2 h. Cell numbers in the lower chamber were analyzed by flow cytometry in presence of Accucheck counting beads. Graph represents migrated cells (CD4+CD44^hi^IL-23R+/GFP) relative to untreated cells (migration index, mean ± sd, *n* = 8 independent cultures, ***p = 0.0002, ****p < 0.0001). (i) Proposed model of the role of IL-23 signaling pathway in the development of inflammatory diseases. Statistical analysis: (b, c, d, g, h) One-way ANOVA test with Dunnett´s correction for multiple comparisons. (e) One-sample *t* test. (f) *t* test with Welch´s correction. Individual numerical values for quantifications presented in Fig 8 can be found in S8 Data. EAE, experimental autoimmune encephalomyelitis; GFP, Green Fluorescent Protein; IL-23, Interleukin 23; IMQ, Imiquimod; LN, lymph nodes; nTh17, natural Th17; ROCK, Rho-associated protein kinase.

The pathogenic role of IL-23 has been primarily linked to its ability to promote IL-17 and IL-22 production through STAT3 activation and RORγt expression. In this context, ROCK activation has been previously linked to regulation of STAT3 transcriptional activity [36,37]. Thus, we hypothesized if IL-23-mediated ROCK activation could be involved in STAT3 phosphorylation and/or cytokine production in IL-7-expanded Tγδ17 cells. Our experiments showed that ROCK inhibition did not affect IL-23-mediated STAT3 phosphorylation (Fig 8A). Moreover, whereas the interference with the IL-23R signaling pathway using the JAK2 inhibitor AZD1480 blocked the production of IL-17a and IL-22 in response to IL-23, treatment with Y27632 did not alter the production of these pro-inflammatory cytokines (Fig 8B). These experiments offered additional insights about the IL-23 signaling pathway: although both JAK2 and ROCK inhibition blocked IL-23-induced pRLC-S20 (Fig 6A and 6B), ROCK activity was not required to induce pSTAT3-Y705 (Fig 8A). Thus, these data suggested that ROCK activation takes place downstream of JAK2 activation in IL-23 signaling cascade. ROCK activation and RLC phosphorylation are mainly involved in actomyosin remodeling [17]. Hence, we investigated whether IL-23 induced changes in Tγδ17 actomyosin cytoskeleton and thus in cell morphology. Imaging of IL-7-expanded Tγδ17 showed heterogeneous cultures, comprising cells with both polarized and rounded morphology. Moreover, the data showed that IL-23 stimulation induced a 20% to 30% increase in the frequency of Tγδ17 cells with polarized morphology (Fig 8C). In contrast, the treatment with ROCK inhibitor Y27632 drastically reduced the frequency of polarized cells. As polarized cell shape is closely linked to migratory potential, the data suggested that IL-23 increased the fraction of motile Tγδ17 cells. Cell motility is regulated by three main modules: chemokine receptors, integrin-mediated cell adhesion, and actin reorganization [38]. Because pRLC-S20 is widely used as an actin-remodeling marker, we established a migration assay to study the impact of IL-23 on actin cytoskeleton and thus on cell migration. To separate the impact on migration of chemotaxis and integrin-mediated adhesion from the actin remodeling module, we modified conventional transmigration assays and performed the transwell experiments in presence of equal concentrations of IL-23 both in the upper and lower chamber of the transwell, in absence of any exogenous chemotactic or adhesion cues. Remarkably, we found that IL-23 stimulation induced up to a 50% increase in Tγδ17 migration (Fig 8D). Moreover, IL-23-induced migration was abolished in presence of the JAK2 inhibitor AZD1480, demonstrating that sustained IL-23R signaling through JAK2 was required to promote Tγδ17 migration. These results revealed a novel role of IL-23 in the regulation of Tγδ17 migration. Finally, we stimulated Tγδ17 cells with IL-23 and performed the migration assay in the presence of the ROCK inhibitor Y27632. These experiments showed that ROCK inhibition completely abolished the migration of Tγδ17 induced by IL-23. Thus, we have determined that IL-23 promoted Tγδ17 migration through JAK2 and ROCK dependent mechanisms. JAK and ROCK signaling promote actomyosin contractility downstream IL-6 in the context of tumor cells and stromal fibroblast [36], but IL-23 signaling has not been previously linked to actin dynamics nor to cell migration. Thus, we compared IL-23-induced migration to bonafide chemotactic cues in conventional transwell assays, in which chemokines were added only to the lower chamber. As CCR2 and CCR6 have been shown to participate in recruitment of Tγδ17 and Th17 cells to inflamed site [39–41], we determined migration towards the ligands for these chemokine receptors (CCL2 and CCL20, respectively). This comparison showed that the range of IL-23-induced migration was similar to bonafide chemotactic cues for Tγδ17 cells (Fig 8E).

We wanted to examine if IL-23-mediated ROCK activation was required for lymphocyte migration and thus for recruitment to inflammation site. Because direct blockage of IL-23 signaling will have a global impact on inflammation because it will lead to decreased production of pro-inflammatory cytokines, we hypothesized whether interference with ROCK reduce Tγδ17 recruitment to inflamed site. Constant administration of ROCK inhibitors have shown protective effects in inflammatory diseases, including some IL-23-related pathologies [42], but a direct role of ROCK on Tγδ17 recruitment to inflammation site has not been examined. In response to IMQ sensitization, Tγδ17 cells expand in lymph nodes and then migrate to inflamed skin [41]. We adoptively transferred Tγδ17 from IMQ-sensitized animals into recipient animals that were treated with IMQ in the ear skin to provide an inflammation site. Then, we examined the impact of acute inhibition of ROCK on the recruitment of adoptively transferred Tγδ17 cells to the inflamed skin. Mice treated with Y27632 showed a decreased number of transferred Tγδ17 cells in ear skin after 12 h of in vivo migration assay compared to mice treated with control vehicle (Fig 8F, left graph). Remarkably, Tγδ17 migration to distal sites such as inguinal lymph nodes was not impaired by ROCK inhibition, suggesting a selective requirement of ROCK activity for Tγδ17 migration to inflamed tissues (Fig 8F, right graph). These data suggested that IL-23, through the activation of ROCK, could facilitate Tγδ17 migration and recruitment to inflamed sites.

Finally, we examined if IL-23 induced an increase in the migratory potential of nTh17 and iTh17 cells in a ROCK-dependent manner. We performed transwell assays with the nTh17 subpopulation in presence of IL-23 and the ROCK inhibitor. These experiments showed a slight increase of nTh17 migration in presence of IL-23 that did not reach statistical significance, and this increase in migration was abolished in presence of ROCK inhibitor (Fig 8G). The transwell assays performed with iTh17 cells isolated from the EAE model showed a significant increase in cell migration that was blocked upon ROCK inhibition (Fig 8H). Interestingly, ROCK inhibition has been reported to reduce leukocyte infiltration into the central nervous system and to ameliorate disease onset in EAE models [43–45]. Overall, the data presented in Fig 8 showed that IL-23 promoted Tγδ17 and Th17 migration in a ROCK-dependent manner and that ROCK activity was required for T-cell migration to the inflamed site. To summarize, our global phosphoproteomic analysis has uncovered ROCK and RLC as novel components of the IL-23 signaling pathway and revealed a functional role of this signaling cascade in cell migration that could contribute to the development of IL-23 mediated inflammatory diseases (Fig 8I).

## Discussion

The transcriptional program that controls Th17 differentiation and function has been extensively studied using both genome-wide and single-cell genomic approaches [12–16]. Here, we have gone one step further and provided new insights on signaling cascades in Th17 that may be useful for the development of novel therapies for autoimmune and inflammatory diseases. Using global phosphoproteomic analysis of IL-23 response in primary murine Th17 cells, we globally identified approximately 7,000 p-sites, and 3,009 p-sites were consistently identified and quantified in three biological replicates. Of those, IL-23 stimulation induced significant changes in 168 p-sites, widely expanding the current knowledge about the IL-23 signaling cascade. Given the relevance of IL-23 and IL-17-secreting cells in autoimmune and inflammatory diseases, this study is a subject of great interest. In this work, we have validated IL-23-mediated phosphorylation of RLC-S20, a key residue to control the actomyosin cytoskeleton contractility, in two different IL-23-responding subpopulations of pro-inflammatory T cells, the Th17 and Tγδ17 cells. We have determined that IL-23-mediated pRLC-S20 requires the activation of JAK2 and ROCK kinases. Moreover, our work revealed a novel and unexpected role of IL-23 promoting migration of Tγδ17 and Th17 cells through ROCK activation that might facilitate recruitment of pro-inflammatory cells to the inflammation site.

Cell motility is a key feature of the immune system, particularly important for T cells because they are constantly circulating between blood, secondary lymphoid organs, and inflammation sites. In inflammatory pathologies such as EAE or psoriasis, the recruitment of IL-23-responding cells (Tγδ17 and Th17) to inflamed tissues is a critical step for the development of disease [1,41]. Here, we report that IL-23 increases polarized morphology, a key step to coordinate cell movement in response to extracellular cues and promotes Tγδ17 migration through ROCK activation and possibly through pRLC-S20. In addition, we show that ROCK activity is required for Tγδ17 recruitment to inflamed skin and that the increase in cell migration regulated by IL-23 via ROCK activation is conserved in Th17 cells. Thus, the triggering of the IL-23/ROCK/pRLC-20 axis could promote an increase in actomyosin contractibility, joining forces with chemokine receptors and integrin-mediated adhesion to facilitate the recruitment of IL-23-responsive cells to inflammation site. In this context, ROCK activity has been linked to IL-23-related pathologies [42]. Particularly, administration of pharmacological inhibitors of ROCK such as Fasudil [43,44], the Y27632 derivative WAR5 [45], or statins [46] ameliorate disease severity and neuroinflammation in EAE. Moreover, the administration of the oral ROCK inhibitor KD025 to psoriasis vulgaris patients reduced the severity score, epidermal thickness, and T-cell infiltration [47]. Hence, reduced T-cell infiltration correlates with protective effects of ROCK inhibitors, although the complexity of in vivo models prevents the establishment of a direct link between protection and blockage of IL-23-mediated ROCK activation, because ROCK/pRLC-S20 signaling module is also used by integrins, chemokine, and other surface receptors to control cell motility, migration, and even transcription [42]. Our in vitro migration assays provide a system to analyze IL-23 signaling in the absence of other chemotactic or adhesion cues that influence cell migration. In correlation with the increased frequency of polarized cells, our assays clearly show that IL-23 increases the motile capacity of Tγδ17 and Th17 cells in a ROCK-dependent manner. Because the available tools (inhibitors, RNA knockdown, etc.) cannot separate the impact of IL-23 signaling blockage on cytokine production from the effect on cell migration in vivo, the study of the precise contribution of IL-23-induced migration in the context of inflammatory diseases require further research and the development of new tools. In this context, the identification of the molecules linking JAK2 and ROCK activation could generate novel strategies to dissect the different functions of IL-23. ROCK is an essential downstream effector of the Rho family of small guanosine triphosphatases (Rho-GTPases), which activity is regulated by the opposing actions of guanine nucleotide exchange factors (Rho-GEFs), and GTPase-activating proteins (Rho-GAPs) [48]. Interestingly, JAKs have been linked to Rho-GTPases and actin cytoskeleton downstream of chemokine receptors. JAKs are required for T-cell homing to secondary lymphoid organs mediated by chemokine receptors CXCR4 and CCR7 [49,50] and for chemokine-induced integrin affinity maturation, a central step for lymphocyte migration [51]. The proposed molecular mechanisms for JAK-mediated Rho-GTPase activation depend on tyrosine phosphorylation of Rho-GEFs such as Vav1 [51,52] and SOS1 [53]. Dock2, a Rac- and Rho-GEF, is another interesting candidate to mediate JAK/ROCK activation. Dock2 is critical for actin polymerization, maintenance of polarized morphology, and migration of T cells [38,54,55], and our phosphoproteomic study shows IL-23-responsive phosphorylation sites in Dock2 in two biological replicates. An interesting strategy to further dissect IL-23 signaling would be the characterization of JAK2/Tyk2 substrates downstream of IL-23. Of the approximately 7,000 p-sites globally identified in our study, only 113 were tyrosine residues. These numbers reflect that pTyr is a rare event (1%–2% of the Th17 phosphoproteome) compared to pSer or pThr and thus is difficult to detect by unbiased phosphopeptide enrichment workflows. pTyr enrichment protocols can be applied to characterize pTyr events [21], and this strategy can be used to find JAK2 substrates that can act as Rho-GEFs and more importantly, to identify novel components and functions of IL-23 signaling cascade. Regarding the effector molecules downstream of ROCK responsible for the increase in cell migration, the results presented here correlate with IL-23-mediated RLC-S20 phosphorylation, although we cannot exclude the involvement of other ROCK substrates. We have not found evidences of other ROCK substrates such as LIMK or the Ezrin-Radixin-Moesin proteins (ERM) being phosphorylated in response to IL-23 in our phosphoproteomic study; however, we cannot exclude the participation of other ROCK substrates in IL-23-mediated migration.

Our study provides a powerful resource to explore phosphorylation networks in Th17 cells and the IL-23 signaling cascade. In addition to STAT3 phosphorylation, we did not find IL-23-responsive p-sites for key transcription factors of Th17 differentiation such as RORγt or Maf1 [12]. However, we have found IL-23-responsive p-sites in relevant transcription factors for T-cell function that suggest new connections between IL-23 and the regulation of transcriptional activity. For example, different p-sites in the members of the forkhead box family of transcription factors FoxO1 and Foxk1 are up-regulated in response to IL-23. The transcription factors forkhead box O (FoxO) are involved in the integration of growth factor signaling, oxidative stress, and inflammation to control the immune response [56]. FoxO1 transcriptional activity is controlled by phosphorylation, and the IL-23-responsive p-site found in our phosphoproteomics study, the Ser467 residue, has been reported as phosphorylated by MAPK kinases [57]. We have also found IL-23-responsive p-sites in proteins with a relevant function in T-cell signaling cascades. For example, we found up-regulated phosphorylation of a Ser residue in the Linker for activation of T-cells family member 1 (LAT), a well-known participant of the TCR signaling cascade [58]. The adaptor function of LAT is mainly controlled by phosphorylation in Tyr residues, but some reported work suggests additional mechanism of LAT regulation through Ser/Thr phosphorylation [59,60]. In addition to LAT, an up-regulated p-site was found in the Tyrosine-protein phosphatase nonreceptor type 22 (Ptpn22), a phosphatase that has recently emerged as a key protein in the negative regulation of TCR signaling and T-cell effector function [61,62]. Thus, the potential cooperation between IL-23 and TCR signaling cascades is an interesting angle for further research. Recently, a phosphoproteomic study in the human T-cell line Kit225 found that IL-23 promoted Pkm2-Ser37 phosphorylation, linking IL-23 and cellular metabolism [63]. As this residue was not found in our study, we cannot confirm whether IL-23-mediated pPkm2-S37 is conserved in primary Th17 cells, but, all together, these unbiased studies demonstrate the potential of phosphoproteomic approaches to finding unexpected links between cell surface receptors and cellular functions. In addition to offering novel insights into the IL-23 signaling pathway, we provide a comprehensive view of murine Th17 phosphorylation landscape that can be exploited to manipulate the kinase-substrate networks that control effector function.

## Materials and methods

### Ethics statement

Mice breeding and procedures were performed in accordance with national and institutional guidelines for animal care (EU Directive 2010/63/EU for animal experiments). The experimental procedures were approved by the Director General de Medio Ambiente de la Comunidad de Madrid (Approval references: PROEX 269/14 and PROEX 235/17).

### Mice

IL-23R reporter mice with GFP knocked into the *Il23r* locus (*Il23r*-gfp) were kindly provided by Dr. F. Powrie (Oxford, United Kingdom), with the permission of Dr. M. Oukka, who originally generated this mouse strain [25]. Heterozygous mice were used for this study (*Il23r*^wt/gfp^). OT-II TCR transgenic mice expressing a TCR specific for ovalbumin-derived peptide 323–339 (OVA_323-339_) in the context of I-Ab presentation and C57BL/6 were purchased from Jackson Laboratory. Mice were maintained at the Conventional Animal Facility of the School of Medicine of the Universidad Autónoma de Madrid (UAM, ES-28079-0000097) and at the Animal Facility of the Centro de Biología Molecular “Severo Ochoa.”

### Cell isolation, cultures, and treatments

#### OT-II/Th17 differentiation

Lymph nodes (LNs) from 10 to 14-week-old OT-II mice were harvested, pooled, and mechanically disaggregated. Naïve CD4+ T cells were isolated by magnetic sorting (AutoMACS, Miltenyi Biotec) using a CD4 T cell isolation kit (Miltenyi Biotec) according to the manufacturer’s instructions. Biotinylated anti-CD25 (PC61; Tonbo) and anti-CD44 (IM7; BD Pharmingen) were added during the last 5 min for depletion of regulatory and memory T cells; 90% to 95% purity of naïve CD4 cells was routinely obtained. T-cell-depleted and irradiated splenocytes were used as a source of antigen presenting cells. Total splenocytes from OT-II or C57BL/6 mice were incubated with biotinylated anti-CD4 (RM4-5; BD Pharmingen) and anti-CD8 (clone 53–6.7; Tonbo) antibodies followed by Streptavidin Microbeads incubation (Miltenyi Biotec), and T cells were depleted by magnetic sorting. T-cell-depleted splenocytes received a 25 Gy g-irradiation dose and were mixed with T cells at a 3:1 (APC:T) ratio. Co-cultures were resuspended at 3 × 10^6^ cells/mL in IMDM (Invitrogen) supplemented with L-glutamine (Lonza), 10% (v/v) heat-inactivated fetal bovine serum (FBS; Hyclone, GE Healthcare), penicillin (50 U/ml; Invitrogen), streptomycin (50 mg/ml; Invitrogen), and 2-mercaptoethanol (50 μM; Sigma) (IMDM-10%-2ME). OT-II cognate peptide (OVA_323-339_; SQAVHAAHAEINEAGR; Invivogen) was added at 5μg/mL, together with Th17 polarizing cytokines: TGFβ (3 ng/mL; Prepotec), IL-6 (50 ng/mL; Prepotec), IL-1β (10 ng/mL; Prepotec), IL-21 (10 ng/mL; Prepotec), IL-23 (10 ng/mL; Miltenyi Biotec). After 4 days, freshly T-cell-depleted and irradiated splenocytes, OVA_323-339_ peptide, and Th17 polarizing cytokines were added to the cultures. On day 8, cells were stimulated with phorbol 12,13-dibutyrate (PDBu; 20 ng/mL; Calbiochem) and Ionomycin (Io; 0.5ng/mL; Calbiochem) for 4 h in presence of Golgi-Plug (BD Biosciences) or Golgi-Plug alone and processed for detection of intracellular cytokines by flow cytometry.

#### Anti-CD3/CD28 Th17 and Th0 differentiation

Naïve CD4 cells from C57BL/6 mice were isolated as described above and stimulated with plate-bound anti-CD3 (2C11; 5 μg/mL; Tonbo) and anti-CD28 (37.51; 2 μg/mL; eBioscience) in IMDM-10%-2ME supplemented with Th17 polarizing cytokines for 4 to 5 days. For Th0 generation, cells were stimulated as above in absence of polarizing cytokines.

#### TCRγδ cultures

Pooled LN from 5 to 10 mice (12 to 24 weeks) were mechanically disaggregated and incubated with a biotin-coupled anti-TCRγδ antibody (GL3; BD Pharmingen) followed by incubation with Streptavidin Microbeads. TCRγδ cells were isolated by positive selection using AutoMacs or MS columns (Miltenyi Bitotec); 50% to 80% purity of TCRγδ cells was typically obtained with this method. Isolated TCRγδ cells were cultured in IMDM-10%-2ME supplemented with IL-7 (5 ng/mL; Prepotec) for 5 to 10 days. IL-7 was refreshed every 3 days. Cells were treated with the following inhibitors: JAK2 inhibitors AZD1480 (5 μM; SelleckChem) and TG101348 (2 μM; SelleckChem), ROCK inhibitor Y27632 (10–20 μM; Tocris), and MLCK inhibitor ML7 (10 μM; Tocris).

#### nTh17

Pooled LN and spleens from 15 to 20 mice (12–24 weeks) were mechanically disaggregated and incubated with anti-CD4 (RM4-5; BD Pharmingen) followed by Streptavidin Microbeads incubation (Miltenyi Biotec) for CD4 T cells enrichment by magnetic selection. Afterwards, nTh17 were sorted as CD4+CD44^hi^IL-23R/GFP+ using a BD FACSAria Fusion (BDBiosciences). Sorted cells were cultured in IMDM-10%-2ME supplemented with IL-7 for 7 days before stimulation with IL-23. IL-7 was refreshed every 3 days.

### Quantitative real-time PCR

RNA extracted using Ribospin kit (GeneAll Biotechnology Co.) was reverse-transcribed with a qScript cDNA synthesis kit (Quanta BioSciences), according to the manufacturer’s protocols. Quantitative PCR was performed in 96-well plates in triplicate using the Power SYBR Green PCR mix and a StepOne Plus Real-Time PCR System (Applied Biosystems). The abundance of hypoxanthine phosphoribosyl-transferase 1 (*Hprt*) mRNA was used for normalization. The following primers were used:

*Hprt* forward, 5'-TGATCAGTCAACGGGGGACA-3',

*Hprt* reverse, 5'-TTCGAGAGGTCCTTTTCACCA-3',

*Rorc* forward, 5'-GACCCACACCTCACAAATTGA-3',

*Rorc* reverse, 5'-AGTAGGCCACATTACACTGCT-3',

*Il23r* forward, 5'-GCCAAGAAGACCATTCCCGA-3',

*Il23r* reverse, 5'-TCAGTGCTACAATCTTCTTCAGAGGAC-3'.

### Sample preparation for phosphoproteomics

Eight-day OT-II/Th17 cultures were subjected to a Ficoll gradient to remove cell debris and washed twice in IMDM-10% to remove remaining cytokines. Cells were cultured in IMDM-10%-2ME without polarizing cytokines for 4 h prior to stimulation with IL-23 at 10 ng/mL for 30 min. Phosphoproteomics was performed using approximately 60 × 10^6^ cells in the three biological replicates (30 × 10^6^ per condition). After stimulation, cells were pelleted, washed twice in ice-cold HBSS, and lysed at 50 × 10^6^ cells/mL in 8 M urea, 50 mM Tris-HCl (pH 8), supplemented with protease and phosphatase inhibitor tablets (Complete Mini-EDTA and PhosphoStop; Roche). Lysates were vigorously shaken for 15 min/RT, sonicated, and shaken for additional 15 min/RT. The amount of extracted protein was determined by BCA assay (Pierce). Approximately 1 mg of protein was obtained per condition in each biological replicate. The protein lysates were then reduced (10 mM DTT; Sigma-Aldrich) and alkylated (50 mM iodoacetamide; Sigma-Aldrich) prior to overnight digestion at 37°C with trypsin (1:80 trypsin:protein, w/w; Promega). Digested proteins were desalted using C18 Sep-Pak cartridges (Waters) and subjected to dimethyl labeling.

### Dimethyl labeling

Dimethyl labeling was performed on C18 Sep-Pak cartridges following on-column labeling protocol [64]. Briefly, 4.5 mL of 50 mM sodium phosphate buffer pH 7.5 (1 mL 50 mM NaH_2_PO_4_ + 3.5 mL 50 mM Na_2_HPO_4_; Merck), mixed with 250 μL 4% formaldehyde (v/v) and 250 μL of 0.6 M cyanoborohydride (NaBH_3_CN; Sigma-Aldrich), was used per sample/label. The control condition (untreated) was labeled with light formaldehyde (CH_2_O; Sigma-Aldrich), and the IL-23-stimulated condition was labeled with intermediate formaldehyde (CD_2_O; Sigma-Aldrich). Differentially labeled eluates were mixed at a 1:1 ratio (Ctrl:IL-23) and subjected to fractionation by HILIC.

### HILIC and phosphopeptide enrichment

Dimethyl labeled (1:1 mixed conditions) from three biological replicates were fractionated using Ultimate 3000 HPLC (Thermo Scientific) equipped with a 4.6 × 250- mm TSKgel Amide-80 5-μm particle column (Tosoh Biosciences). The buffers used for the separation were 0.1% TFA (HILIC buffer A) and 99.9% acetonitrile, 0.1% TFA (HILIC buffer B). The peptide samples were resuspended in 80% HILIC buffer B and injected onto the HILIC column. The chromatography was performed using the following elution gradient: 80% B held for 20 min followed by 80% B to 60% B in 40 min and finally 0% B for 10 min at a flow rate of 0.4 mL/min. In total, 16 fractions were collected per biological replicate and subjected to phosphopeptide enrichment using titanium dioxide (TiO_2_, Titansphere, GL Science) as previously described [20,23]. Briefly, TiO_2_ spheres were prepared at 50 mg/mL, and 25 μL (1.25 mg TiO2) were added to each sample fraction, incubated for 10 min/RT to bind phosphopeptides. Samples were washed and eluted with 200 μL 0.4 M NH_4_OH followed with 200 μL 0.2 M NH_4_OH/50% acetonitrile and then dried using a speedvac (Genevac).

### Liquid chromatography–mass spectrometry

Phosphopeptide samples were resuspended in 1% formic acid and separated by nanoscale C18 reverse-phase liquid chromatography (Ultimate 3000 RSLC nano system, Thermo Scientific). The buffers used for chromatography were HPLC Buffer A (2% acetonitrile, 0.1% formic acid), HPLC Buffer B (80% acetonitrile, 0.08% formic acid), and HPLC Buffer C (0.1% formic acid). Samples were washed onto the column with HPLC Buffer C and eluted with the following buffer gradient: 2% B (0–3 min), 2% to 40% B (3–128 min), 40% to 98% B (128–130 min), 98% B (130–150 min), 98% to 2% B (150–151 min), and equilibrated in 2% B (151–180 min) at a flow rate of 0.3 μL/min. The eluting peptide solution was automatically electrosprayed into the coupled Linear Trap Quadrupole (LTQ)–Orbitrap mass spectrometer (LTQ-Orbitrap Velos Pro; Thermo Scientific) using an Easy-Spray nanoelectrospray ion source (Thermo Scientific). The mass spectrometers were operated in positive ion mode and were used in data-dependent acquisition modes. A full scan (FT-MS) was acquired at a target value of 1,000,000 ions with resolution R = 60,000 over a mass range of 335 to 1,800 amu. The fifteen most intense ions were selected for fragmentation in the LTQ Orbitrap Velos. Fragmentation in the LTQ was induced by collision-induced dissociation (CID) with a target value of 10,000 ions. For accurate mass measurement, the "lock mass" function (lock mass = 445.120024 Da) was enabled for MS scan modes. To improve the fragmentation of phosphopeptides, the multistage activation algorithm in the Xcalibur software was enabled for each MS/MS spectrum using the neutral loss values of 97.98, 48.99, 32.66, and 24.49 m/z units. Former target ions selected for MS/MS were dynamically excluded for 45 s. General mass spectrometric conditions were as follows: spray voltage, 1.8 to 2.5 kV; no sheath and auxiliary gas flow; ion transfer tube temperature, 250°C; normalized collision energy (35%) using wide band activation mode for MS2. The isolation width was set to 2 amu for IT-MS/MS. Ion selection thresholds were 5,000 counts for MS2. An activation of q = 0.25 and activation time of 10 ms were applied in MS2 acquisitions. The fill time for FTMS was set to 500 ms and for ITMS to 100 ms.

### Data processing

Raw mass spectrometry data were processed in MaxQuant version 1.5.8.3 [65,66] using the following search parameters: a MS tolerance of 20 ppm, MS/MS tolerance of 0.5 Da, and full trypsin specificity. Two missed cleavages were permitted. Protein N-terminal acetylation, oxidation of methionine, glutamine to pyroglutamate conversion, deamidation (NQ), and phosphorylation of serine, threonine and tyrosine were set as variable modifications. Carbamidomethylation of cysteine was set as a fixed modification. The minimum peptide length for identification was set to 6 amino acids. The match between run function was enabled. Proteins were mapped to the Uniprot/Swiss-Prot mouse protein database (retrieved on July 6, 2017), containing 16,894 mouse complete proteome entries. False discovery rates (FDRs) of 0.01 were based on hits against a reversed sequence database and calculated at the level of peptides, proteins, and modification sites. Prior to statistical analysis, the outputs from MaxQuant were filtered to remove known contaminants and reverse sequences. The distribution of differential dimethyl labeling ratios was normalized within MaxQuant at the peptide level so that the median of log_2_ ratios is zero [65]. The mass spectrometry proteomics data have been deposited to the ProteomeXchange Consortium via the PRIDE [67] partner repository with the dataset identifier PXD016633.

### Bioinformatic analysis and statistical tools

For functional enrichment and canonical signaling pathway analysis, the different subsets of proteins were analyzed using Ingenuity Pathway Analysis (Qiagen) against the Ingenuity Knowledge Base for *Mus musculus* as model organism. Perseus version 1.5.8.5 software was used to map identified phosphorylation sites with kinase motifs and to annotate the identified phosphoproteins with Gene Ontology (GO) biological processes (BP) and molecular functions (MF) [68]. Statistical analysis of the dimethyl ratio changes (linear value) was performed using a multiple *t* test with an FDR-5% in Graphad Prism version 6. For p-sites identified in multiple biological replicates, the averaged dimethyl ratio of the replicates was used to represent the data. Heatmap of top IL-23-induced changes was generated using Morpheus (https://software.broadinstitute.org/morpheus). Graphad Prism version 6 was used for statistical analysis. The statistical test used for each figure is indicated in the figure legends.

### Western blot

Protein samples from the biological replicates used for phosphoproteomic analysis were lysed in 8 M urea as described above. Th17 cells were lysed at 20 × 10^6^/mL in RIPA buffer (100 mM HEPES [pH 7.4], 150 mM NaCl, 1% NP40, 0.1% SDS, 0.5% sodium deoxycho- late, 10% glycerol, 1 mM EDTA, 1 mM EGTA, 1 mM TCEP (Pierce), and protease and phosphatase inhibitors [Roche]). Protein samples were mixed with NuPAGE LDS sample buffer (Life Technologies) supplemented with TCEP as reducing agent (25 mM; Sigma-Aldrich). Protein lysates were separated by SDS-PAGE polyacrylamide gel electrophoresis and then transferred to nitrocellulose membrane (Amersham) using standard conditions (Mini-PROTEAN tetra cell system; Bio-Rad). Membranes were blocked with 5% (w/v) nonfat dried skimmed milk powder in phosphate-buffered saline (PBS) containing 0.2% Tween 20. Membranes were probed with the following primary antibodies: pSTAT3-Y705, pSTAT3-S727, STAT3, RLC (all purchased from Cell Signaling Technologies), pRLC-S19/S20 (Rockland Inc.), SCM1 (Bethyl Laboratories) following manufacturer´s recommendations. Primary antibodies were detected using peroxidase-conjugated secondary antibodies (Goat anti-Rabbit-Ig and Goat anti-Mouse-Ig, Thermo Scientific), and chemiluminescence detected with the ImageQuant LAS-4000 imaging system (Fujifilm).

### Imiquimod treatment

*Il23r*-gfp reporter mice were treated with 5% Imiquimod on shaved and depilated back and ear skin for 5 days (50 mg/day; Aldara; Meda Pharma). Skin draining LN (cervical, axillary, brachial, and inguinal) were pooled and processed for flow cytometry analysis. For cytokine production ex vivo, total lymph node cells were stimulated with PDBu (20 ng/mL), Ionomycin (0.5 ng/mL) for 4 h in presence of GolgiPlug or Golgi-Plug alone and processed for detection of intracellular cytokines by flow cytometry. For ex vivo analysis of RLC phosphorylation, total lymph nodes were stimulated overnight in presence or absence of IL-23 (10 ng/mL) and Y27632 (10 μM).

### EAE induction

Mice were injected subcutaneously at two sites with 100 μL of an emulsion of complete Freund´s adjuvant (Sigma) containing 150 μg Myelin Oligodendrocyte Glycoprotein peptide (MOG_35–55_; Sigma) and 1 mg heat-killed M. tuberculosis strain H37Ra (BD). Mice received 200 ng Pertussis toxin from *Bordetella pertussis* (Sigma) intraperitoneally on the day of immunization and 2 days later; 10 to 12 days later, before disease onset, LN and spleens were harvested, mashed, and CD4 T cells were enriched by positive magnetic selection using anti-CD4 (RM4-5; BD Pharmingen) followed by Streptavidin Microbeads incubation (Miltenyi Biotec). Afterwards, iTh17 cells were sorted as CD4+CD44^hi^IL-23R/GFP+. Sorted cells were cultured in IMDM-10%-2ME supplemented with IL-7 for 7 days before restimulation with IL-23.

### Flow cytometry

For dead cell exclusion, cells were incubated with Ghost Dye-Red780 or Dye-Violet510 following manufacturer´s instructions (Tonbo Biosciences), supplemented with Fc block (2.4G2; BD Biosciences) for 30 min/ice prior to antibody staining. For surface staining, cells were washed once in staining solution (PBS, 0.5% FBS, 0.5% bovine serum albumin, and 0.01% sodium azide) and incubated for 20 min/ice using manufacturer’s suggested antibody dilutions in staining solution. Antibodies used against surface targets: CD8 (53–6.7; Tonbo), CD4 (RM4-5; Tonbo), TCRβ (H57-597; Tonbo), TCRγδ (GL3; Biolegend), CD44 (IM7; Tonbo), CD27 (LG.3A10; Biolegend), IL-7R α (SB/199; BD Pharmingen), IL-2R α (PC61; BD Pharmingen), IL-1R1 (35F5; BD Horizon). Fluorochrome-coupled versions of these antibodies were used (fluorochromes used: FITC, PE, APC, PerCP–Cy5.5, PE-Cy7, APC-H7, Horizon V450, BV421). In some stainings, biotin-coupled antibodies followed by fluorochrome-coupled streptavidin were used.

For intracellular staining of cytokine production, after cell surface staining cells were fixed for 20 min/RT (IC Fixation buffer; eBioscience) and incubated with IFNγ (XMG1.2; BD Pharmingen), IL-17a (TC11-18H10; BD Pharmingen and eBio17B7; eBioscience), or IL-22 (Poly5164; Biolegend) diluted in permeabilization buffer (eBioscience), following manufacturer´s instructions. RORγt (AFKJS-9; eBioscience) intracellular staining was performed using FoxP3/Transcription Factor Staining set (eBioscience) following manufacturer´s instructions. For pSTAT3-Y705 staining, after surface staining cells were fixed 30 min/ice, washed, and permeabilized in 90% methanol 30 min/ice. Cells were then washed and incubated in staining solution with anti-pSTAT3-Y705 (D3A7; Cell Signaling) for 30 min/RT, washed twice, and stained with Alexa 647–conjugated anti-rabbit antibody (Jackson ImmunoResearch). For pRLC staining, after surface staining cells were fixed 30 min/ice, washed, and incubated for 30 min/RT with pRLC-S19/S20 (Rockland Inc.) diluted in Permeabilization buffer (eBioscience), washed twice, and stained with Alexa 647–conjugated anti-rabbit antibody (Jackson ImmunoResearch). Samples were acquired on a FACSCanto II flow cytometer with DIVA software and on a FACSCalibur (BD Biosciences) and analyzed with FlowJo software (Tree Star). Cells were gated according to their forward scatter and side scatter profile, and dead cells excluded based on their staining with the viability dye. Tγδ17 cells were electronically gated as CD3+ TCRγδ+ CD44^hi^ and IL-23R/GFP+ when *Il23r*-gfp mice were used. For pSTAT3-Y705 and pRLC-S20, the MFI was normalized to the untreated condition to compare different experiments.

### shRNAs, lentiviral production, and cell transduction

shRNA against ROCK1 cloned into pLKO.1 lentiviral vector (NM_009071/TRCN0000022903; Sigma MISSION shRNA Library) was subcloned into the LV-GFP lentiviral vector as a *SphI*-*SacII* fragment. LV-GFP vector was a gift from Elaine Fuchs (Addgene plasmid #25999). Restriction enzymes were obtained from New England Biolabs. For lentiviral particles production, LV-GFP and LV-GFP-shROCK1 were transiently transfected in HEK293 cells together with the packaging vector psPAX2 and the envelope vector pMD2.G (gifts from Didier Trono; Addgene plasmids #12260 and #12259, respectively) using jetPEI Transfection reagent (Polyplus). Next day, culture supernatants were replaced with fresh media. 48 h and 72 h after transfection, culture supernatants were harvested and concentrated by ultracentrifugation (26.000 rpms/2 h/4°C). Concentrated lentiviral particles were kept at −80°C until use. To test the ROCK1 knockdown efficiency of the shRNA, the mouse fibroblast NIH3T3 cell line was transduced with lentiviral particles in presence of Polybrene (5 μg/mL; Sigma); 5 days later, NIH3T3 cells were processed for western blot analysis of ROCK1 expression. Anti-ROCK1 antibody (BD Transduction Laboratories) was kindly provided by Dr. J. Millán. IL-7-expanded Tγδ17 cells (day 3) were stimulated for 18 h with IL-23 prior to transduction with lentiviral particles in presence of Polybrene (10 μg/mL). Culture plates were spun at 650*g* for 1 h. Next day, cells were washed and resuspended in fresh medium containing IL-7; 7 days later, cells were stimulated with IL-23 and transduction efficiency and pRLC-S20 levels were determined by flow cytometry. HEK293 and NIH3T3 cell lines were maintained in DMEM (Invitrogen) supplemented with L-glutamine, 10% (v/v) FBS, penicillin (50 U/mL) and streptomycin (50 mg/mL).

### Imaging

IL-7-expanded Tγδ17 sorted as CD27 negative were plated into wells precoated with mixture of fibronectin (20 μg/mL, Sigma) and Poly-L-Lys (1 μg/mL, Sigma), and allowed to settle in presence or absence of 10 μM ROCK inhibitor Y27632 for 1 h at 37°C. Bright-field images were acquired in an Axiovert200 (Zeiss) coupled to a sCMOS camera with a 20x dry objective. The number of cells with polarized morphology was manually quantified by two independent observers (100–200 cells were counted per condition and experiment).

### Transmigration assays

Migration assays were performed using 3.0-μm transwell Permeable Supports (CoStar). 0.1 × 10^6^ IL-7-expanded TCRγδ cells, stimulated or not with IL-23 10 ng/mL overnight, were placed in transwell inserts in duplicates in 100 μL of media (IMDM-10%-2ME + 5ng/mL IL-7), in presence or absence of IL-23 (10 ng/mL). The lower chamber was filled with media alone, IL-23, or IL-23 plus the indicated pharmacological inhibitors. For conventional chemotactic assays, 0.1 × 10^6^ IL-7-expanded TCRγδ cells well placed in transwell inserts in duplicates in 100 μL of media, and the lower chamber was filled with 600 μL of media alone, CCL2 (100 ng/mL; Prepotec) or CCL20 (500 ng/mL; CCL20). The number of migrated cells in the lower chamber was determined after 6 h by flow cytometry. For migration assays with sorted nTh17 and iTh17 cells, 0.1 × 10^6^ cells were placed in transwell inserts and 10 μL of AccuCheck Counting Beads (ThermoFisher) were added to the bottom chamber; 2 h later, cells in bottom chamber were collected and stained with CD4 and CD44 before flow cytometry analysis. The migration index was obtained normalizing number of cells in lower chamber to the untreated condition.

### Adoptive transfer and homing to inflamed skin

To obtain Tγδ17 cells for adoptive transfer, ear and back skin of *Il23r*-gfp reporter mice were treated with 5% Imiquimod for 5 days (50 mg/day; Aldara; Meda Pharma). Skin draining LN (cervical, axillary, brachial, and inguinal) were pooled, labeled with CellTrace Violet (5 μM; Invitrogen), and incubated with IL-23 (10 ng/mL) in presence or absence of Y27632 (20 μM) for 6 h before adoptive transfer (10 to 15 × 10^6^ cells per animal) by retro-orbital injection to recipient animals. Recipient ear skin was previously treated for 5 days with Imiquimod (10 mg/ear). Prior to adoptive transfer, recipients received two intraperitoneal doses of Y27632 (10 mg/kg, 8 h apart); 12 h after transfer, ear skin and LN from recipient mice were harvested and processed for flow cytometry analysis. LN cells were mechanically disaggregated. Ears were split in two halves, cut into pieces, and digested for 45 min/37°C in IMDM containing Liberase TM (83 μg/mL; Roche), DNase I (100 μg/mL; Roche) and Collagenase IV (0.5 mg/mL; Sigma). Undigested skin pieces were further subjected to tissue disruption using 7-mm stainless steel beads (Qiagen) and a TissueLyser LT (20 oscilations/5 min; Qiagen). Accucheck counting beads (Life Technologies) were added for accurate counting of adoptively transferred cells before processing the samples for flow cytometry analysis. In LN, Tγδ17 cells were gated as CD3+TCRγδ+CD44hi. In skin, the Tγδ17 cells were gated as dermal TCRγδ cells (CD3+TCRγδintCD44hi).

## Supporting information

S1 Table Th17 phosphoproteome. List of all identified p-sites, presented as protein (Uniprot ID), protein name, gene name, modified amino acid (amino acid), modified position (position), sequence window, normalized ratio IL-23/Ctrl for the three biological replicates (Exp. 1, Exp. 2 and Exp.3), averaged ratio IL-23/Ctrl (linear and log2 values), number of experiments in which the p-sites have been identified (N exp.), MS intensity (intensity), Perseus motif analysis annotation and Perseus GO, and KEGG pathway Perseus annotations. The file includes one tab with the consistent p-sites found in the three biological replicates including the results of the multiple t test FDR-5% significance (Discovery FDR-5% and Adjusted P value), a second tab with the normalized data used in the study, and a third tab with the raw data obtained from Maxquant analysis. Ctrl, untreated control; FDR, false discovery rate; GO, gene ontology; IL-23, Interleukin 23; KEGG, Kyoto Encyclopedia of Genes and Genomes; MS, mass spectrometry; p-site, phosphorylation site.
(XLSX)

S2 Table IPA functional enrichment analysis of Th17 phosphoproteome. List of enriched molecular and cellular functions determined by IPA for the proteins with consistent p-sites identified in the three biological replicates, presented as: category, significance (*p*-value) and proteins included in the indicated category (molecules). IPA, Ingenuity Pathway Analysis; p-site, phosphorylation site
(XLSX)

S3 Table IL-23-regulated phosphoproteome in Th17 cells. List of 168 phosphosites with significant changes using a multiple *t* test FDR-5%, presented as in S1 Table. The file includes one tab for p-sites up-regulated, and a second tab for down-regulated p-sites. FDR, false discovery rate; IL-23, Interleukin 23; p-site, phosphorylation site
(XLSX)

S4 Table IPA functional enrichment analysis of IL-23-regulated phosphoproteome in Th17 cells. List of enriched categories determined by IPA for proteins with significant IL-23-induced changes, presented as category, significance (*p*-value), and proteins included in the indicated category (molecules). The file includes one tab with the Molecular and Cellular Functions analysis and a second tab with the Canonical Pathway analysis. IL-23, Interleukin 23; IPA, Ingenuity Pathway Analysis
(XLSX)

S1 Fig Pairwise comparison of biological replicates. (a) Normalized ratio distribution for the individual biological replicates. Inset numbers indicate the number of phosphosites and protein groups identified and quantified in the individual biological replicates. (b) Pairwise comparison of biological replicates. Venn diagrams show the number and percentage of overlapping p-sites identified in the three biological replicates, and graphs below compare the ratio IL-23/Ctrl (log_2_ value) for the overlapping p-sites. Inset number indicates the correlation coefficient R. Individual numerical values for quantifications presented in S1 Fig can be found in S9 Data. Ctrl, untreated control; IL-23, Interleukin 23; p-site, phosphorylation site
(TIF)

S2 Fig Expression markers in Tγδ17 cells. (a) Lymph node cells from untreated (Ctrl) or IMQ-sensitized *Il23r*-gfp reporter mice were stimulated ex vivo with PDBu/Io or left untreated for 6 h before assessing IL-17a production by flow cytometry. Representative histograms show IL-17a production in Tγδ17 cells (gated as CD3+TCRγδ+CD44hi), and inset number represent the percentage of IL-17a+ cells in the indicated gate. Graph shows the percentage of IL-17a-producers among Tγδ17 cells (mean ± sd, *n* = 4–8 mice). (b) Representative contour plot of IL-7Rα and IL-23R/GFP expression in CD3+TCRγδ+lymph node cells from *Il23r*-gfp reporter mice. Graph shows IL-7Rα MFI in TCRγδ+ IL-23R/GFP+ cells, relative to IL-7Rα MFI in TCRγδ+ GFP- cells (mean ± sd, *n* = 7 mice). (c) TCRγδ cells were isolated from *Il23r*-gfp reporter mice and cultured for 5 days in presence of IL-7. Graph shows the number of TCRγδ+CD44hi cells after 5 days of culture with IL-7, normalized to cell number at day 0 (mean ± sd, *n* = 11 independent cell cultures). (d) Representative contour plots of IL-2Rα and IL-1R1 expression, plotted against CD44 expression, in IL-7-expanded TCRγδ cells (*n* = 3 independent cell cultures). Individual numerical values for quantifications presented in S2 Fig can be found in S10 Data. Ctrl, untreated control; GFP, green fluorescent protein; IL-23, Interleukin 23; IMQ, Imiquimod; MFI, mean of fluorescence intensity; PDBu/Io, Phorbol 12,13-dibutyrate/Ionomycin
(TIF)

S3 Fig IL-17a production in nTh17 and iTh17. (a) Total lymph node cells or spleens from *Il23r*-gfp reporter mice were stimulated ex vivo with PDBu/Io in presence of Golgi-Plug or left unstimulated for 6 h before assessing IL-17a production by flow cytometry. Representative contour plots show CD44 and IL-17a expression, and inset numbers represent the percentage of IL-17a+ cells in the indicated gates. Graph represents the percentages of IL-17a producers among the CD4 population in lymph nodes and spleens (mean ± sd, *n* = 4–5). (b) Total lymph node cell from wild type (*Il23r*^wt/wt^) and *Il23r*-gfp reporter mice (*Il23r*^gfp/wt^) were stimulated with PDBu/Io in the presence of Golgi-Plug or left unstimulated for 6 h before assessing IL-17a production by flow cytometry. Representative contour plots show IL-23R (GFP) and IL-17a expression, inset numbers represent the percentage of cells in each quadrant gate. (c) EAE was induced in *Il23r*-gfp reporter mice. 12 days later, lymph node cells and spleens were stimulated ex vivo with PDBu/Io in the presence of Golgi-Plug or left unstimulated for 6 h before assessing IL-17a production by flow cytometry. Representative contour plots show CD44 and IL-17a expression in spleens, and inset numbers represent the percentage of IL-17a+ cells in the indicated gates. Graph represents the percentage of IL-17a producers among the CD4 population in lymph nodes and spleens (mean ± sd, *n* = 3). (d) EAE was induced in *Il23r*-gfp reporter mice. 12 days later, iTh17 were sorted as CD4+CD44^hi^IL-23R+ from lymph nodes and spleens and cultured for 7 days in presence of IL-7. Graph represents cell numbers, normalized to numbers at d0 (mean ± sd, *n* = 9 independent cultures) (e) IL-7-expanded iTh17 were stimulated with PDBu/Io in the presence of Golgi-Plug or left unstimulated for 4 h before assessing IL-17a production by flow cytometry. Graph represents the percentage of IL-17a producers among the CD4 population (mean ± sd, *n* = 5–8). Individual numerical values for quantifications presented in S3 Fig can be found in S11 Data. EAE, experimental autoimmune encephalomyelitis; GFP, green fluorescent protein; IL-23, Interleukin 23; iTh17, induced Th17; nTh17, natural Th17; PDBu/Io, Phorbol 12,13-dibutyrate/Ionomycin.
(TIF)

S1 Data Individual numerical values underlying quantifications in Fig 1. (XLSX)

S2 Data Individual numerical values underlying quantifications in Fig 2. (XLSX)

S3 Data Individual numerical values underlying quantifications in Fig 3. (XLSX)

S4 Data Individual numerical values underlying quantifications in Fig 4. (XLSX)

S5 Data Individual numerical values underlying quantifications in Fig 5. (XLSX)

S6 Data Individual numerical values underlying quantifications in Fig 6. (XLSX)

S7 Data Individual numerical values underlying quantifications in Fig 7. (XLSX)

S8 Data Individual numerical values underlying quantifications in Fig 8. (XLSX)

S9 Data Individual numerical values underlying quantifications in S1 Fig. (XLSX)

S10 Data Individual numerical values underlying quantifications in S2 Fig. (XLSX)

S11 Data Individual numerical values underlying quantifications in S3 Fig. (XLSX)

S1 Raw images Western blot raw images for Fig 1G. (TIF)

S2 Raw images Western blot raw images for Fig 3B. (TIF)

S3 Raw images Western blot raw images for Fig 4E. (TIF)

S4 Raw images Western blot raw images for Fig 6E. (TIF)

## Abbreviations

BP: biological process
CID: collision-induced dissociation
CK2: Casein kinase 2
CMGC: kinase family including cyclin-dependent kinases (CDKs), mitogen-activated protein kinases (MAP kinases), glycogen synthase kinases (GSK) and CDK-like kinases
Ctrl: untreated control
EAE: experimental autoimmune encephalomyelitis
Erk1/2: Extracellular signal-regulated kinases ½
ERM: Ezrin-Radixin-Moesin proteins
FDR: false discovery rate
FoxO: forkhead box O
GFP: Green fluorescent protein
GO: gene ontology
GRK1: G-protein-coupled receptor kinase 1
HILIC: hydrophilic interaction liquid chromatography
Hist1h1c: histone H1C
Hsp90ab1: heat shock protein 90 alpha family
IL-17: interleukin 17
IL-23: Interleukin 23
ILC3: Type 3 innate lymphoid cells
IMQ: Imiquimod
IPA: Ingenuity Pathway Analysis
iTh17: induced Th17
JAK: Janus family of tyrosine kinase
LAT: Linker for activation of T-cells family member 1
LN: lymph node
LTQ: Linear Trap Quadrupole
MAPK: Mitogen-Activated Protein Kinase
MF: molecular function
MFI: mean of fluorescence intensity
MYPT-1: myosin Phosphatase-Targeting Subunit 1
nTh17: natural Th17
OVA: ovalbumin
PDBu/Io: Phorbol 12,13-dibutyrate/Ionomycin
PKA: Protein kinase A
PKC: Protein kinase C
pRLC-S20: phosphorylated myosin regulatory light chain in Serine20 residue
p-site: phosphorylation site
Ptpn22: Tyrosine-protein phosphatase nonreceptor type 22
RLC: myosin regulatory light chain
ROCK: Rho-associated protein kinase
RORgt: RAR Related Orphan Receptor C
shROCK1: shRNAs against ROCK1
STAT: Signal Transducer and Activator of Transcription
TCRγδ: TCR gamma/delta cells
Tγδ17: IL-17-secreting TCR gamma/delta cells
