# Supplementary figures and images for: IL-23 signaling regulation of pro-inflammatory T-cell migration uncovered by phosphoproteomics
Source: PLoS Biol. 2020 Mar 23;18(3):e3000646. doi: 10.1371/journal.pbio.3000646 (PMC7117768; doi:10.1371/journal.pbio.3000646)

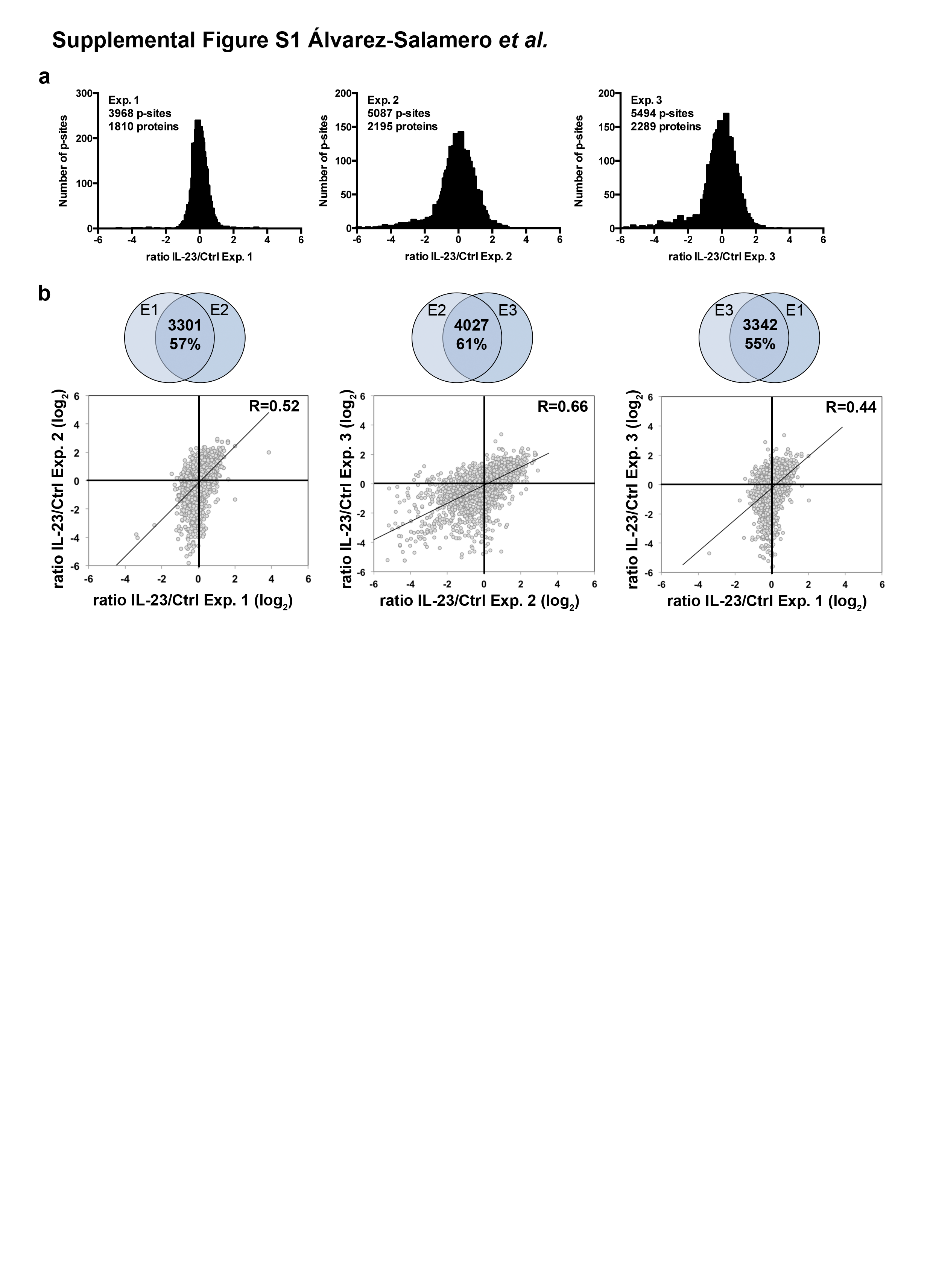

Supplement: S1 Fig — (a) Normalized ratio distribution for the individual biological replicates. Inset numbers indicate the number of phosphosites and protein groups identified and quantified in the individual biological replicates. (b) Pairwise comparison of biological replicates. Venn diagrams show the number and percentage of overlapping p-sites identified in the three biological replicates, and graphs below compare the ratio IL-23/Ctrl (log2 value) for the overlapping p-sites. Inset number indicates the correlation coefficient R. Individual numerical values for quantifications presented in S1 Fig can be found in S9 Data. Ctrl, untreated control; IL-23, Interleukin 23; p-site, phosphorylation site (TIF) [file pbio.3000646.s005.tif]

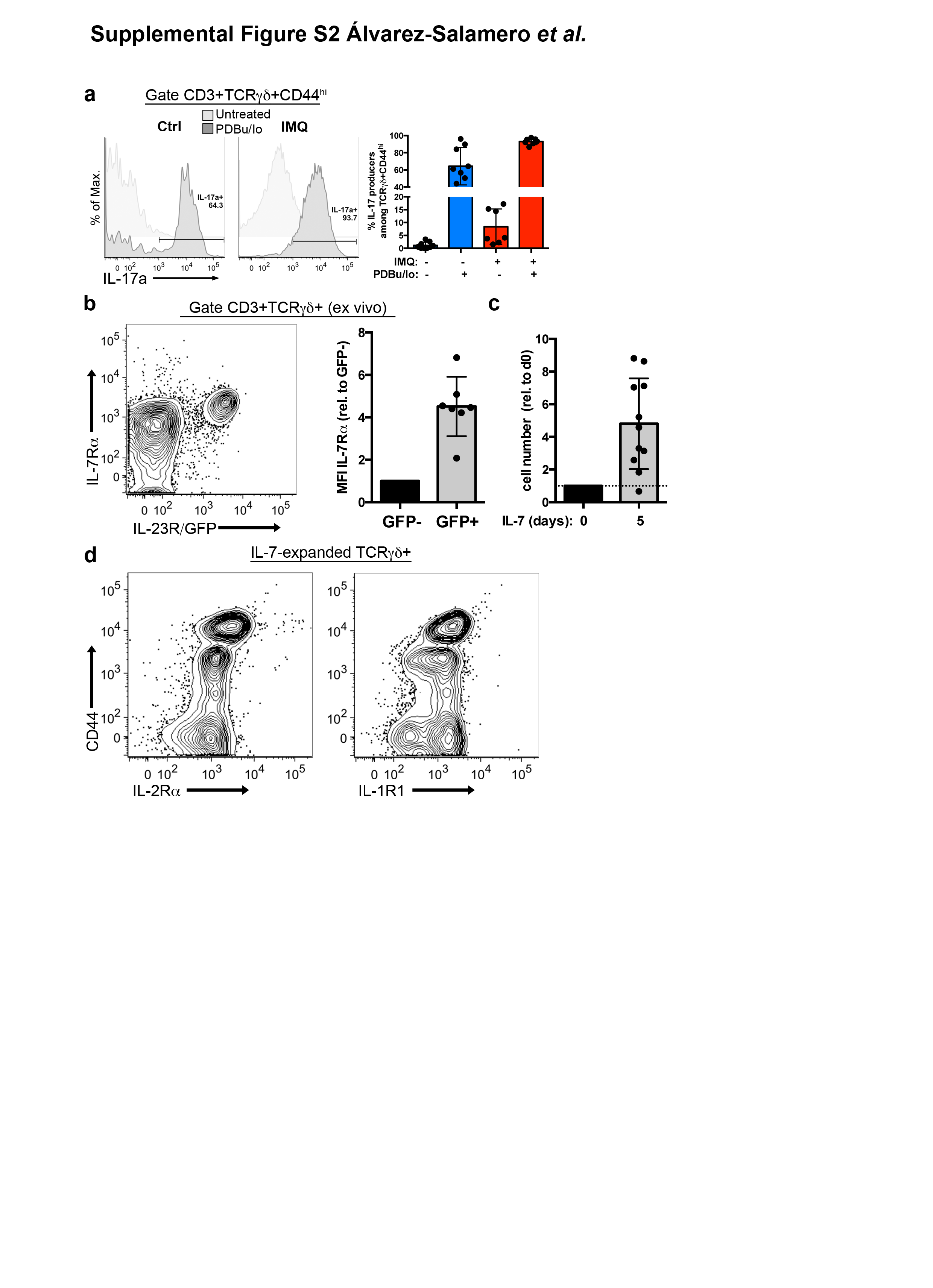

Supplement: S2 Fig — (a) Lymph node cells from untreated (Ctrl) or IMQ-sensitized Il23r-gfp reporter mice were stimulated ex vivo with PDBu/Io or left untreated for 6 h before assessing IL-17a production by flow cytometry. Representative histograms show IL-17a production in Tγδ17 cells (gated as CD3+TCRγδ+CD44hi), and inset number represent the percentage of IL-17a+ cells in the indicated gate. Graph shows the percentage of IL-17a-producers among Tγδ17 cells (mean ± sd, n = 4–8 mice). (b) Representative contour plot of IL-7Rα and IL-23R/GFP expression in CD3+TCRγδ+lymph node cells from Il23r-gfp reporter mice. Graph shows IL-7Rα MFI in TCRγδ+ IL-23R/GFP+ cells, relative to IL-7Rα MFI in TCRγδ+ GFP- cells (mean ± sd, n = 7 mice). (c) TCRγδ cells were isolated from Il23r-gfp reporter mice and cultured for 5 days in presence of IL-7. Graph shows the number of TCRγδ+CD44hi cells after 5 days of culture with IL-7, normalized to cell number at day 0 (mean ± sd, n = 11 independent cell cultures). (d) Representative contour plots of IL-2Rα and IL-1R1 expression, plotted against CD44 expression, in IL-7-expanded TCRγδ cells (n = 3 independent cell cultures). Individual numerical values for quantifications presented in S2 Fig can be found in S10 Data. Ctrl, untreated control; GFP, green fluorescent protein; IL-23, Interleukin 23; IMQ, Imiquimod; MFI, mean of fluorescence intensity; PDBu/Io, Phorbol 12,13-dibutyrate/Ionomycin (TIF) [file pbio.3000646.s006.tif]

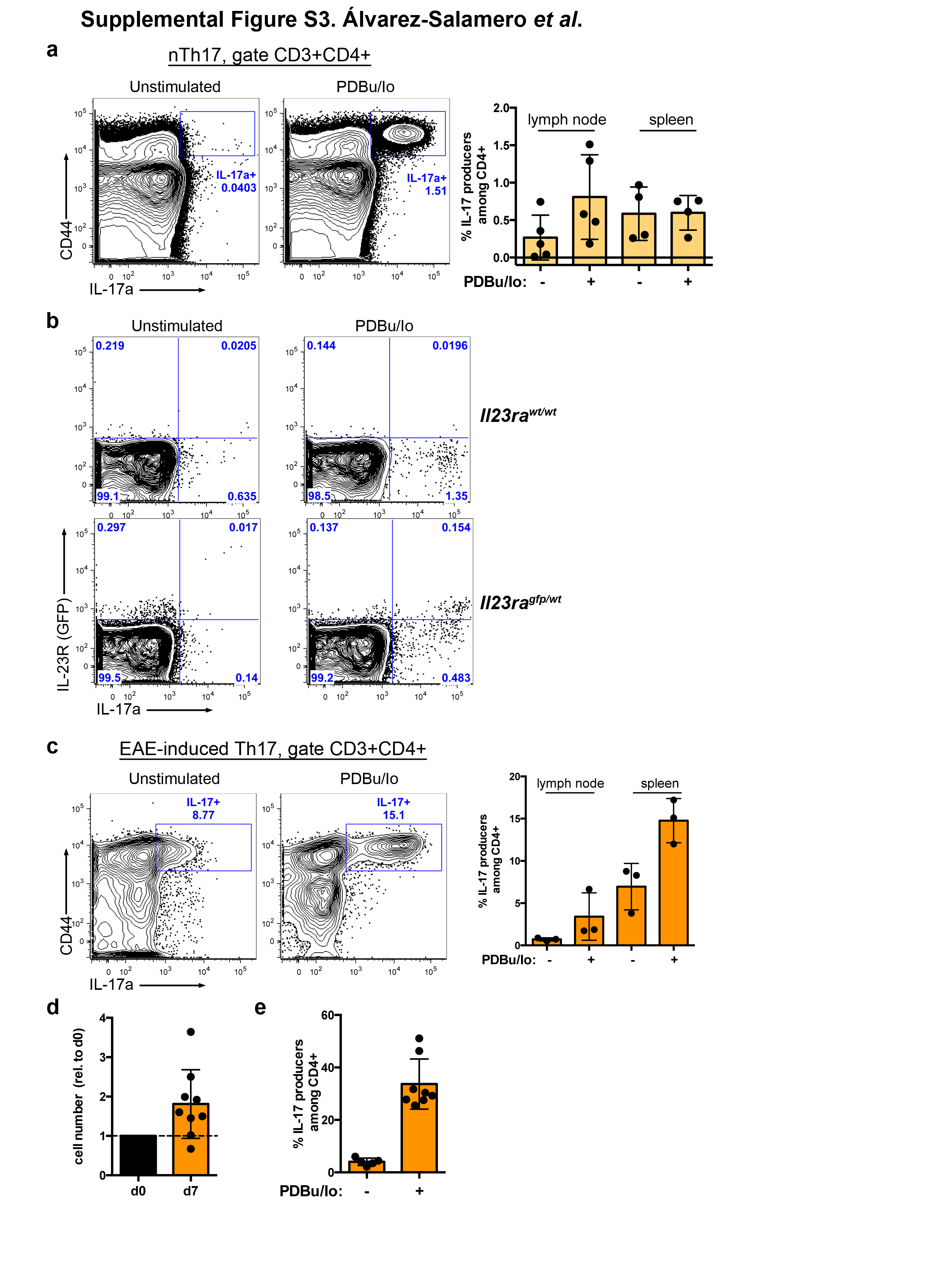

Supplement: S3 Fig — (a) Total lymph node cells or spleens from Il23r-gfp reporter mice were stimulated ex vivo with PDBu/Io in presence of Golgi-Plug or left unstimulated for 6 h before assessing IL-17a production by flow cytometry. Representative contour plots show CD44 and IL-17a expression, and inset numbers represent the percentage of IL-17a+ cells in the indicated gates. Graph represents the percentages of IL-17a producers among the CD4 population in lymph nodes and spleens (mean ± sd, n = 4–5). (b) Total lymph node cell from wild type (Il23rwt/wt) and Il23r-gfp reporter mice (Il23rgfp/wt) were stimulated with PDBu/Io in the presence of Golgi-Plug or left unstimulated for 6 h before assessing IL-17a production by flow cytometry. Representative contour plots show IL-23R (GFP) and IL-17a expression, inset numbers represent the percentage of cells in each quadrant gate. (c) EAE was induced in Il23r-gfp reporter mice. 12 days later, lymph node cells and spleens were stimulated ex vivo with PDBu/Io in the presence of Golgi-Plug or left unstimulated for 6 h before assessing IL-17a production by flow cytometry. Representative contour plots show CD44 and IL-17a expression in spleens, and inset numbers represent the percentage of IL-17a+ cells in the indicated gates. Graph represents the percentage of IL-17a producers among the CD4 population in lymph nodes and spleens (mean ± sd, n = 3). (d) EAE was induced in Il23r-gfp reporter mice. 12 days later, iTh17 were sorted as CD4+CD44hiIL-23R+ from lymph nodes and spleens and cultured for 7 days in presence of IL-7. Graph represents cell numbers, normalized to numbers at d0 (mean ± sd, n = 9 independent cultures) (e) IL-7-expanded iTh17 were stimulated with PDBu/Io in the presence of Golgi-Plug or left unstimulated for 4 h before assessing IL-17a production by flow cytometry. Graph represents the percentage of IL-17a producers among the CD4 population (mean ± sd, n = 5–8). Individual numerical values for quantifications presented in S3 Fig can [file pbio.3000646.s007.tif]

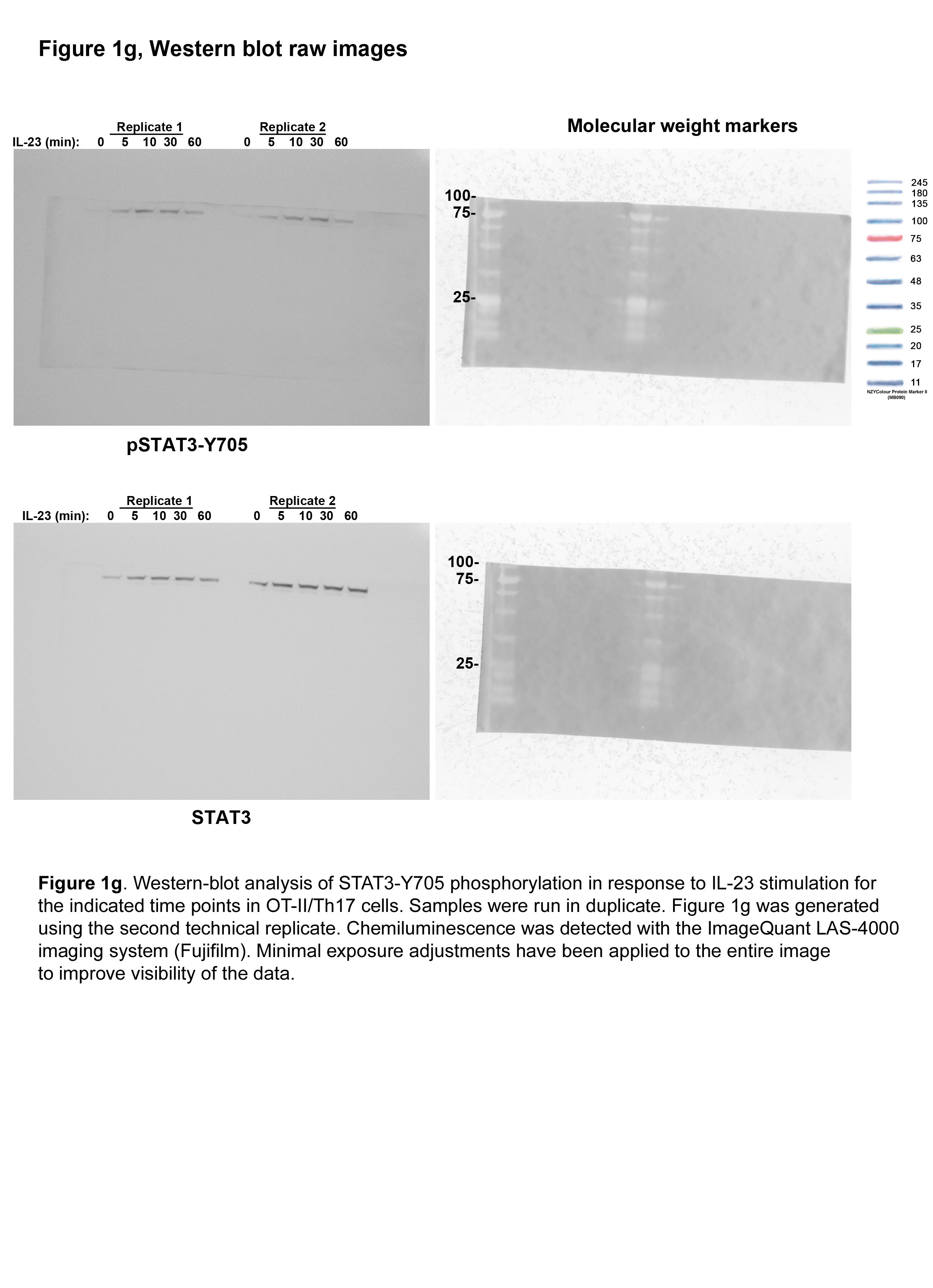

Supplement: S1 Raw images — (TIF) [file pbio.3000646.s019.tif]

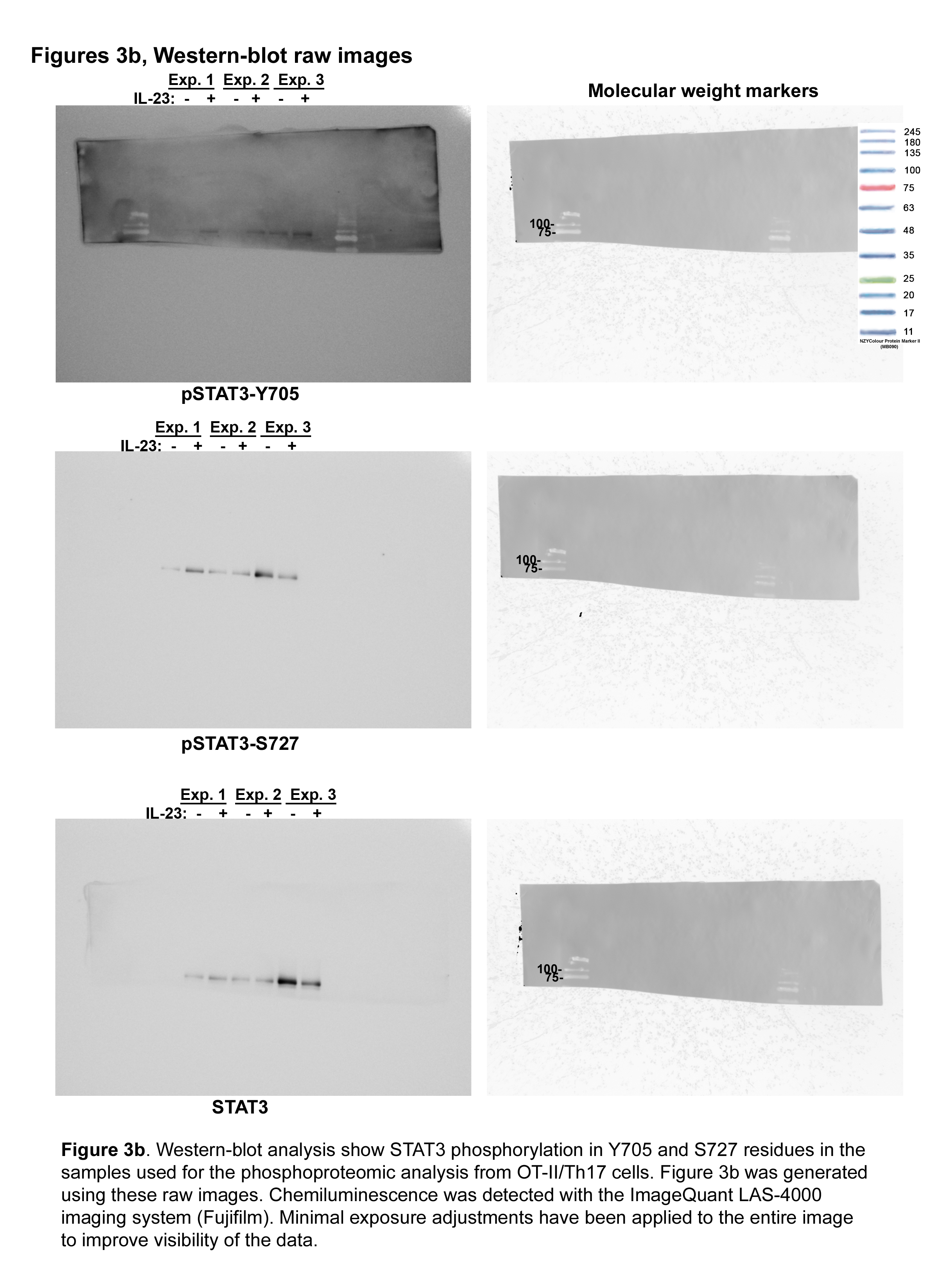

Supplement: S2 Raw images — (TIF) [file pbio.3000646.s020.tif]

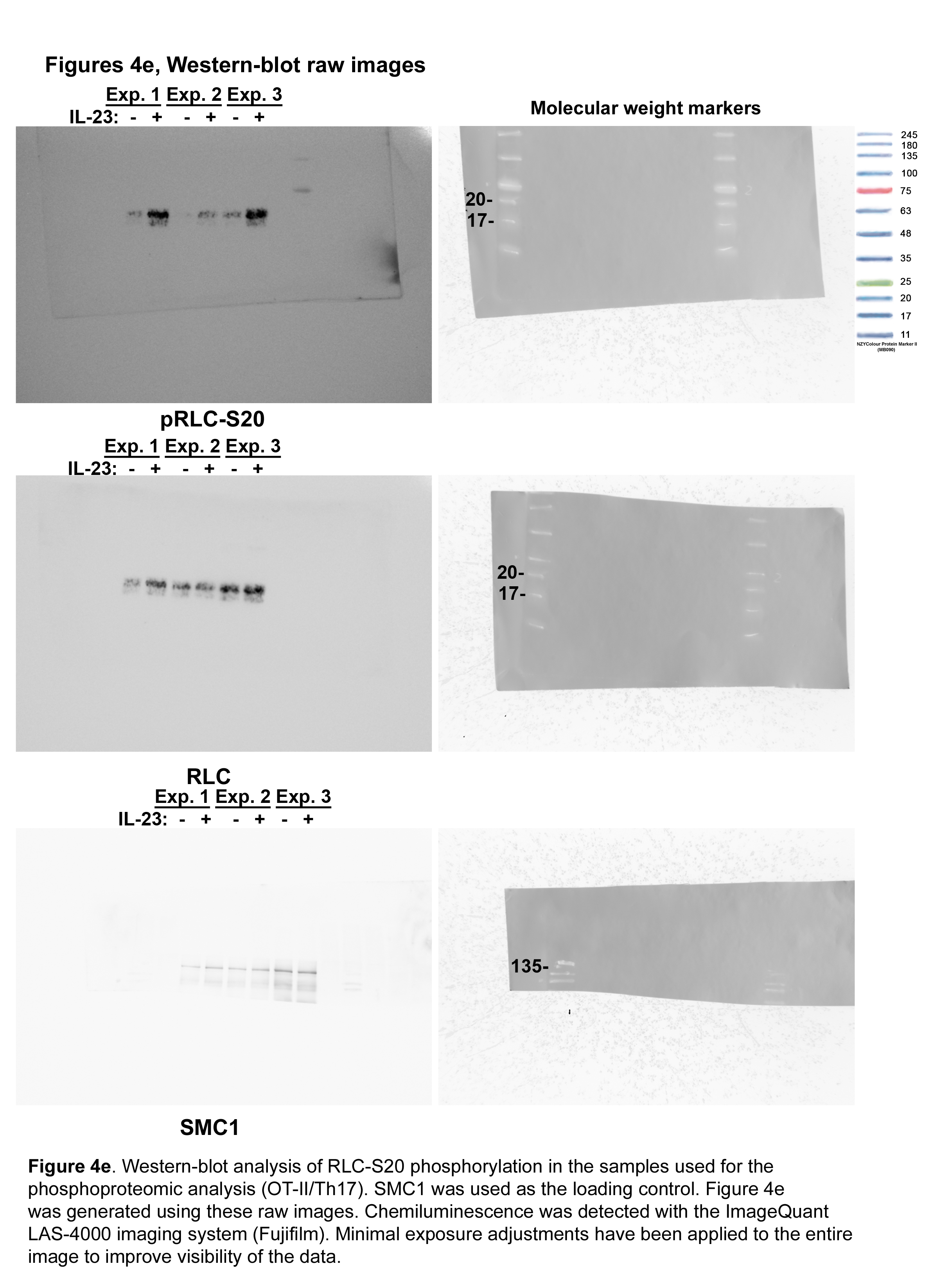

Supplement: S3 Raw images — (TIF) [file pbio.3000646.s021.tif]

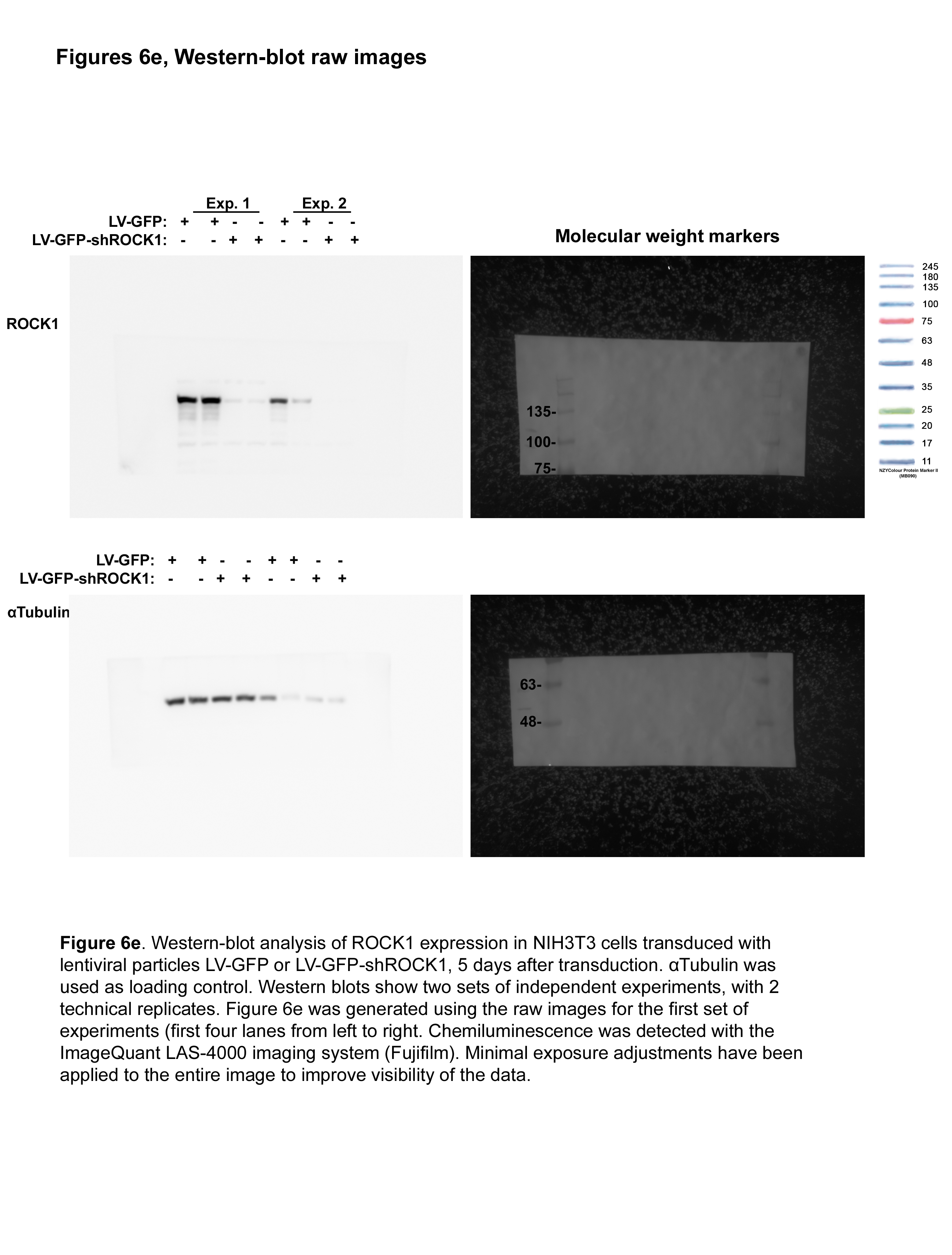

Supplement: S4 Raw images — (TIF) [file pbio.3000646.s022.tif]
